# Supplementary material for: Impact of anthropometry training and feasibility of 3D imaging on anthropometry data quality among children under five years in a postmortem setting
Source: PLoS One. 2023 Sep 28;18(9):e0292046. doi: 10.1371/journal.pone.0292046 (PMC10538800; doi:10.1371/journal.pone.0292046)
Supplement: Attachment — Submitted filename: PONE-D-23-01635_R1_reviewer.pdf [file pone.0292046.s007.pdf]

PLOS ONE

Impact of anthropometry training and feasibility of 3D imaging on anthropometry data quality among children under five years in a postmortem setting

--Manuscript Draft--

Manuscript Number:	PONE-D-23-01635R1
Article Type:	Research Article
Full Title:	Impact of anthropometry training and feasibility of 3D imaging on anthropometry data quality among children under five years in a postmortem setting
Short Title:	Anthropometry in the postmortem setting
Corresponding Author:	Parminder Suchdev<br>Emory University School of Medicine<br>Atlanta, Georgia UNITED STATES
Keywords:	Nutrition, Anthropometry, Child mortality
Abstract:	BACKGROUND: The Child Health and Mortality Prevention Surveillance Network (CHAMPS) identifies causes of under-5 mortality in high mortality countries.<br>OBJECTIVE: To address challenges in postmortem nutritional assessment, we evaluated the impact of anthropometry training and the feasibility of 3D imaging on data quality within the CHAMPS Kenya site.<br>DESIGN: Staff were trained using World Health Organization (WHO)-recommended manual anthropometry equipment and novel 3D imaging methods to collect postmortem measurements. Following training, 76 deceased children were measured in duplicate and were compared to measurements of 75 pre-training deceased children. Outcomes included measures of data quality (standard deviations of anthropometric indices and digit preference scores (DPS)), precision (absolute and relative technical errors of measurement, TEMs or rTEMs), and accuracy (Bland-Altman plots). WHO growth standards were used to produce anthropometric indices. Post-training surveys and in-depth interviews collected qualitative feedback on measurer experience with performing manual anthropometry and ease of using 3D imaging software.<br>RESULTS: Manual anthropometry data quality improved after training, as indicated by DPS. Standard deviations of anthropometric indices exceeded limits for high data quality when using the WHO growth standards. Reliability of measurements post-training was high as indicated by rTEMs below 1.5%. 3D imaging was highly correlated with manual measurements; however, on average 3D scans overestimated length and head circumference by 1.61 cm and 2.27 cm, respectively. Site staff preferred manual anthropometry to 3D imaging, as the imaging technology required adequate lighting and additional considerations when performing the measurements.<br>CONCLUSIONS: Manual anthropometry was feasible and reliable postmortem in the presence of rigor mortis. 3D imaging may be an accurate alternative to manual anthropometry, but technology adjustments are needed to ensure accuracy and usability.
Order of Authors:	Priya Mehta Gupta, MPH
	Kasthuri Sivalogan
	Richard Oliech
	Eugene Alexander
	Jamie Klein
	O.Yaw Addo
	Dickson Gethi
	Victor Akelo
	Dianna Blau
	Parminder Suchdev

Powered by Editorial Manager® and ProduXion Manager® from Aries Systems Corporation

Response to Reviewers:	Full response to editor and reviewer comments has been uploaded.
Additional Information:	
Question	Response
**Financial Disclosure**<br><br>Enter a financial disclosure statement that describes the sources of funding for the work included in this submission. Review the submission guidelines for detailed requirements. View published research articles from *PLOS ONE* for specific examples.<br><br>This statement is required for submission and **will appear in the published article** if the submission is accepted. Please make sure it is accurate.<br><br>**Unfunded studies**<br>Enter: *The author(s) received no specific funding for this work.*<br><br>**Funded studies**<br>Enter a statement with the following details:<br>• Initials of the authors who received each award<br>• Grant numbers awarded to each author<br>• The full name of each funder<br>• URL of each funder website<br>• Did the sponsors or funders play any role in the study design, data collection and analysis, decision to publish, or preparation of the manuscript?<br>• **NO** - Include this sentence at the end of your statement: *The funders had no role in study design, data collection and analysis, decision to publish, or preparation of the manuscript.*<br>• **YES** - Specify the role(s) played.<br><br>* typeset	Sources of Support: This work was funded by grant OPP1126780 from the Bill & Melinda Gates Foundation.<br><br>The funder participated in discussions of study design and data collection. They did not participate in the conduct of the study; the management, analysis, or interpretation of the data; preparation, review, or approval of the manuscript; or decision to submit the manuscript for publication.
**Competing Interests**<br><br>Use the instructions below to enter a competing interest statement for this submission. On behalf of all authors, disclose any competing interests that could be perceived to bias this work—acknowledging all financial support and any other relevant financial or non-financial competing interests.	I have read the journal's policy and the authors of this manuscript have the following competing interests: Eugene Alexander holds an ownership position in Body Surface Translations and therefore has a financial interest in the success of the 3D testing device described in this study. Data were blinded and not shared with Mr. Alexander until completion of draft manuscript.<br><br>This does not alter our adherence to PLOS ONE policies on sharing data and materials.<br>Additional disclosure: The findings and conclusions in this report are those of the

This statement is required for submission and **will appear in the published article** if the submission is accepted. Please make sure it is accurate and that any funding sources listed in your Funding Information later in the submission form are also declared in your Financial Disclosure statement.<br><br>View published research articles from *PLOS ONE* for specific examples.<br><br>**NO authors have competing interests**<br><br>Enter: *The authors have declared that no competing interests exist.*<br><br>**Authors with competing interests**<br><br>Enter competing interest details beginning with this statement:<br><br>*I have read the journal's policy and the authors of this manuscript have the following competing interests: [insert competing interests here]*<br><br>* typeset	authors and do not necessarily represent the official position of the Centers for Disease Control and Prevention.
**Ethics Statement**<br><br>Enter an ethics statement for this submission. This statement is required if the study involved:<br><br>• Human participants<br>• Human specimens or tissue<br>• Vertebrate animals or cephalopods<br>• Vertebrate embryos or tissues<br>• Field research<br><br>Write "N/A" if the submission does not require an ethics statement.<br><br>General guidance is provided below. Consult the submission guidelines for detailed instructions. **Make sure that all**	Ethics committees or institutional review boards overseeing investigators at each site and at Emory University (Emory IRB#: 00091706) approved the generic and site-specific protocols, as appropriate. The Centers for Disease Control and Prevention formally relied on the Emory University IRB for review of the overall protocol and on appropriate site ethical review committees where CDC staff were directly engaged at the site. Details of ethical protocols are available at https://champshealth.org/protocols/.

Powered by Editorial Manager® and ProduXion Manager® from Aries Systems Corporation

**information entered here is included in the Methods section of the manuscript.**<br><br>**Format for specific study types**<br><br>**Human Subject Research (involving human participants and/or tissue)**<br>• Give the name of the institutional review board or ethics committee that approved the study<br>• Include the approval number and/or a statement indicating approval of this research<br>• Indicate the form of consent obtained (written/oral) or the reason that consent was not obtained (e.g. the data were analyzed anonymously)<br><br>**Animal Research (involving vertebrate animals, embryos or tissues)**<br>• Provide the name of the Institutional Animal Care and Use Committee (IACUC) or other relevant ethics board that reviewed the study protocol, and indicate whether they approved this research or granted a formal waiver of ethical approval<br>• Include an approval number if one was obtained<br>• If the study involved *non-human primates*, add *additional details* about animal welfare and steps taken to ameliorate suffering<br>• If anesthesia, euthanasia, or any kind of animal sacrifice is part of the study, include briefly which substances and/or methods were applied<br><br>**Field Research**<br><br>Include the following details if this study involves the collection of plant, animal, or other materials from a natural setting:<br>• Field permit number<br>• Name of the institution or relevant body that granted permission	
**Data Availability**<br><br>Authors are required to make all data underlying the findings described fully available, without restriction, and from the time of publication. PLOS allows rare exceptions to address legal and ethical	Yes - all data are fully available without restriction

concerns. See the PLOS Data Policy and FAQ for detailed information.<br><br>A Data Availability Statement describing where the data can be found is required at submission. Your answers to this question constitute the Data Availability Statement and **will be published in the article**, if accepted.<br><br>**Important:** Stating 'data available on request from the author' is not sufficient. If your data are only available upon request, select 'No' for the first question and explain your exceptional situation in the text box.<br><br>Do the authors confirm that all data underlying the findings described in their manuscript are fully available without restriction?	
**Describe where the data may be found in full sentences. If you are copying our sample text, replace any instances of XXX with the appropriate details.**<br><br>• If the data are **held or will be held in a public repository**, include URLs, accession numbers or DOIs. If this information will only be available after acceptance, indicate this by ticking the box below. For example: *All XXX files are available from the XXX database (accession number(s) XXX, XXX.*).<br>• If the data are all contained **within the manuscript and/or Supporting Information files**, enter the following: *All relevant data are within the manuscript and its Supporting Information files.*<br>• If neither of these applies but you are able to provide **details of access elsewhere**, with or without limitations, please do so. For example:<br><br>*Data cannot be shared publicly because of [XXX]. Data are available from the XXX Institutional Data Access / Ethics Committee (contact via XXX) for researchers who meet the criteria for access to confidential data.*	The minimal anonymized data set necessary to replicate our study findings has been included as Supporting Information

The data underlying the results presented in the study are available from (include the name of the third party and contact information or URL).<br>• This text is appropriate if the data are owned by a third party and authors do not have permission to share the data.<br><br>* typeset	
Additional data availability information:	Tick here if the URLs/accession numbers/DOIs will be available only after acceptance of the manuscript for publication so that we can ensure their inclusion before publication.

Powered by Editorial Manager® and ProduXion Manager® from Aries Systems Corporation

Manuscript
Click here to access/download;Manuscript;PlosOne Revised manuscript Clean_rev.docx

Impact of anthropometry training and feasibility of 3D imaging on anthropometry data quality among children under five years in a postmortem setting

Priya M. Gupta[1], Kasthuri Sivalogan[1], Richard Oliech[2], Eugene Alexander[3], Jamie Klein[4], O. Yaw. Addo[1,6], Dickson Gethi[5], Victor Akelo[5], Dianna M. Blau[6], Parminder S. Suchdev[1,4,6*]

1. Nutrition and Health Sciences Program, Laney Graduate School, Emory University, Atlanta, GA, USA
2. Kenya Medical Research Institute, Kisumu, Kenya
3. Body Surface Translations, Inc. (BST), Athens, GA, USA
4. Emory University School of Medicine, Atlanta, GA, USA
5. US Centers for Disease Control and Prevention-Kenya, Kisumu and Nairobi, Kenya
6. US Centers for Disease Control and Prevention, Atlanta, GA, USA

Conflicts of interest:
Eugene Alexander holds an ownership position in Body Surface Translations and therefore has a financial interest in the success of the 3D testing device described in this study. Data were blinded and not shared with Mr. Alexander until completion of draft manuscript.

Additional disclosure: The findings and conclusions in this report are those of the authors and do not necessarily represent the official position of the Centers for Disease Control and Prevention.

Data Sharing: Data described in the manuscript, code book, and analytic code will be made available upon request pending [e.g., application and approval, payment, other].

***Corresponding Author**
Parminder S Suchdev
1599 Clifton Rd NE
Atlanta, GA 30322
psuchde@emory.edu

Running Title: Anthropometry in the postmortem setting

Abstract

BACKGROUND: The Child Health and Mortality Prevention Surveillance Network (CHAMPS) identifies causes of under-5 mortality in high mortality countries.

OBJECTIVE: To address challenges in postmortem nutritional assessment, we evaluated the impact of anthropometry training and the feasibility of 3D imaging on data quality within the CHAMPS Kenya site.

DESIGN: Staff were trained using World Health Organization (WHO)-recommended manual anthropometry equipment and novel 3D imaging methods to collect postmortem measurements. Following training, 76 deceased children were measured in duplicate and were compared to measurements of 75 pre-training deceased children. Outcomes included measures of data quality (standard deviations of anthropometric indices and digit preference scores (DPS)), precision (absolute and relative technical errors of measurement, TEMs or rTEMs), and accuracy (Bland-Altman plots). WHO growth standards were used to produce anthropometric indices. Post-training surveys and in-depth interviews collected qualitative feedback on measurer experience with performing manual anthropometry and ease of using 3D imaging software.

RESULTS: Manual anthropometry data quality improved after training, as indicated by DPS. Standard deviations of anthropometric indices exceeded limits for high data quality when using the WHO growth standards. Reliability of measurements post-training was high as indicated by rTEMs below 1.5%. 3D imaging was highly correlated with manual measurements; however, on average 3D scans overestimated length and head circumference by 1.61 cm and 2.27 cm, respectively. Site staff preferred manual anthropometry to 3D imaging, as the imaging technology required adequate lighting and additional considerations when performing the measurements.

CONCLUSIONS: Manual anthropometry was feasible and reliable postmortem in the presence of rigor mortis. 3D imaging may be an accurate alternative to manual anthropometry, but technology adjustments are needed to ensure accuracy and usability.

Introduction

Malnutrition is estimated to contribute to approximately half of under-5-mortality (U5M) [1-3]. Malnutrition is also a major cause of morbidity as malnutrition plays a critical role in child neurodevelopment and health across the life course [2-4]. Reliable assessment tools for malnutrition are essential to reflect individual status, measure biological function, and predict health outcomes [5-7]. In children, inadequate growth is defined according to anthropometric measurements (length, weight, head and mid-upper arm circumference) that fall below 2 standard deviations of the normal sex-specific weight-for-length (wasting), length-for-age (stunting), and weight-for-age (underweight) [7]. Despite the importance of accurate anthropometry to detect early signs of malnutrition and monitor child growth, health facilities routinely use non-standardized anthropometric equipment, and as a result, measurements are often inaccurate [8]. Inaccurate measurements can lead to spurious classification of malnutrition in both individuals and populations[9].

In addition to the challenges of procuring and using standard anthropometric measurement tools, anthropometric measurements are subject to human error and are particularly difficult to collect among young children as children are easily distressed, have difficulty staying still, and may be unable to meet the requirements (i.e. ability to lie down or stand up) for manual anthropometry [10-12]. Anthropometric measurements are particularly challenging in hospitalized settings or in medically complex patients due to medical equipment that may impede taking measurements (e.g., intravenous lines or feeding tubes), severe illness, or limitations in mobility. These children are also at highest risk of malnutrition [8, 13]. Additionally, qualitative findings from a quality improvement study in a children's hospital found that, wooden height-length measuring boards (ShorrBoard®, Weigh and Measure, LLC, Maryland USA) were considered to be "*heavy, cumbersome to assemble, frightening to patients, and required pre-planning and coordination between clinical staff with busy schedules and competing priorities*" [8]. Lastly, in field settings, the weight of the board may impede transportation and movement within the field and lack of standardization and maintenance of anthropometric equipment across study sites may contribute to poor data quality and misclassification [10, 11]. The post-mortem setting is another environment in which manual anthropometry may be challenging. Morgue capacity, rigor mortis, and edema can impact the quality and accuracy of measurements [14]. To our knowledge, no research has been conducted on the feasibility of using gold-standard anthropometric assessment in the postmortem setting.

The Child Health and Mortality Prevention Surveillance (CHAMPS) network is a multi-site surveillance system which strives to identify and understand the causes of under-5-mortality (U5M) in seven surveillance sites in sub-Saharan Africa and South Asia through detailed cause of death attribution with the use of high-quality postmortem anthropometrics, tissue samples, clinical abstraction, verbal autopsy, and the ability to integrate data from site-specific health and demographic surveillance systems (HDSS) [15, 16]. A recent analysis of the postmortem anthropometric data in CHAMPS suggested that nearly 90% of cases 1-59 months had evidence of undernutrition (stunting, wasting, or underweight) [17]. Given these data, it is possible that

malnutrition is directly or indirectly associated with child mortality. However, our understanding of the relationship between malnutrition and mortality may also be hindered by poor anthropometric measurement data quality, including digit preference (e.g. measurement rounding), high percentage of biologically implausible values, and standard deviations for anthropometric indices that exceed acceptable limits, which may lead to misclassification of malnutrition [18-20]. These data quality and precision outcomes may be a result of shortages of standard equipment in CHAMPS sites, lack of training on manual anthropometry, or difficulty in conducting manual anthropometry in the postmortem setting (rigor mortis, poor lighting in morgue facilities).

Our primary objectives were to determine whether manual anthropometry is feasible in the postmortem setting and to quantify the impact of training and standard equipment on data quality. Given the practical challenges of performing manual anthropometry in field and hospital-based settings, various 3D imaging approaches have also been developed to obtain anthropometric measurements. An efficacy study conducted at Emory University found that a 3D imaging software was as accurate as gold-standard manual anthropometry among under-5 children in Atlanta-area daycare centers [10]. However, data are also needed to assess 3D imaging in challenging hospital- or field-based settings. Therefore, our secondary objective was to assess the validity and acceptability of 3D imaging for anthropometric assessment compared to gold-standard manual anthropometry.

Materials and methods

Study site and data collection

This longitudinal quality improvement study took place from October 2018 to September 2019 in the CHAMPS Manyatta, Kenya site located at the Jaramogi Oginga Odinga Teaching and Referral Hospital (JOOTRH). Prior to the training, site staff performed manual anthropometry on 75 deceased children as a routine part of the minimally invasive tissue sampling (MITS) portion of CHAMPS data collection. The MITS procedure is an abridged postmortem examination technique that has been validated for cause of death investigation in low-resource settings, described in detail in an earlier study [21]. Written informed consent was obtained from families as part of the CHAMPS enrollment procedures. The CHAMPS protocol was approved by ethics committees in Kenya and at Emory University, Atlanta, GA, USA. Additional information regarding the ethical, cultural, and scientific considerations specific to inclusivity in global research is included in the Supporting Information.

Upon conclusion of pre-training data collection, a senior nutritionist, pediatrician, and anthropometry expert led and conducted an on-site 1-week training on manual anthropometry and the 3D imaging scanner for 6 staff. Using materials developed by the CDC, WHO and UNICEF, the training on manual anthropometry emphasized best practices for accurate manual measures of length, weight, head circumference (HC) and mid-upper arm circumference (MUAC) measurements using two trained anthropometrists and standard operating procedures [22]. Standard equipment in both sites, including wooden height-length measuring boards

(ShorrBoard®, Weigh and Measure, LLC, Maryland USA), digital scales (Rice Lake Weighing Systems, Inc., Rice Lake, WI), and standard tape measures (Weigh and Measure LLC, Maryland USA), were used to ensure accurate measurement of recumbent length, weight, HC and MUAC, respectively. Staff completed an anthropometry standardization exercise using live children to ensure competence in conducting manual anthropometry. Staff were also trained on proper use the 3D imaging software using dolls and live children; details on the imaging software are provided in earlier studies [10, 23, 24]. Briefly, the AutoAnthro system uses an iPad™ tablet, and a Structure Sensor™ camera attached to the tablet to capture non-personally identifiable anthropometric scan images of the deceased child. Following the training, two trained anthropometrists manually collected anthropometric measurements for 76 cases, with two separate measurements collected per case by different anthropometrists. Additionally, 3D scans were completed in duplicate for each anthropometrist, for a total of 4 scans per case. During data processing, after the completion of data collection, it was identified that the AutoAnthro software settings had been inadvertently altered for a significant number of cases, resulting in a final sample size of 23 cases.

Outcomes of interest

Key outcomes of interest included measures of data quality, precision, and accuracy. Data quality outcomes indicators included digit preference and standard deviations (SD) of anthropometric indices. Digit preference is the examination of a uniform distribution of terminal digits. We also calculated a digit preference score (DPS) to evaluate digit preference [25]. The DPS ranges from 0 to 100. Scores are low in instances of high agreement with the ideal of non-preference of the terminal digits, whereas DPS rises as the measures deviate from a uniform distribution across the terminal digits 0 through 9. In previous studies, a DPS cutoff above 20 was used to define the presence of digit preference [26, 27]. We thus used DPS<20 as acceptable, and DPS≥20 to indicate digit preference was problematic. Previous studies have suggested acceptable standard deviation ranges specifically for data quality among living children [28]. These include 1.10-1.30, 1.00-1.20, 0.85-1.10 for length-for-age (HAZ), weight-for-age (WAZ), and weight-for-length (WLZ) z-scores, respectively. Z-scores for anthropometric indices were produced using the WHO Multicentre Growth Reference Study anthro R package [29].

Technical errors of measurement (TEM) were used to assess measurement precision. Following the training, the site staff performed manual anthropometry in duplicate. It is important to note that this differs from the data collection strategy pre-training in which a single set of measures were taken. As a result, we were only able to calculate TEMs for the data post-training in both sites. TEM express the error margin in anthropometry; they are unitless and allow comparison of errors across measures (e.g., weight, height etc.). Absolute TEMs were calculated using the formula outlined in Equation 1 (**Table 4**). Absolute TEMs can also be transformed into relative TEMs, which express the error as a percentage corresponding to the total average. Relative TEMs (rTEM) were calculated using the formula outlined in Equation 2 (**Table 4**). We used a cutoff of <1.5% rTEM to indicate a skillful anthropometrist [25].

Finally, Bland Altman plots were used to assess the accuracy of the 3D imaging software relative to manual anthropometry following the training and were quantified in the unit of the measure (cm or kg). Spearman correlation coefficients examined the strength of the relationship between scans and manual measures.

Following the study, a short survey was sent to the 6 study participants. The survey collected information on whether the participants believed training on manual anthropometry improved the accuracy of the measurements, whether 3D imaging reduced the time to measure, and asked about the participants preference in measuring using manual anthropometry or the 3D imaging technology. We also conducted a 60-minute in-depth interview with the single lead site technician to collect qualitative feedback on the team's experience with performing manual anthropometry and ease of using the 3D imaging software. All analyses were conducted in R statistical software [30]. Statistical tests were two-sided and evaluated using an alpha level equal to 0.05. Pearson's Chi-Square tests (categorical variables) or t-tests (continuous variables) were used to evaluate differences between pre-intervention and post-interventions groups. The qualitative data were utilized to improve the implementation of manual anthropometric measurements across the CHAMPS Network.

We also conducted a small study in collaboration with the Pediatrics and Pathology departments at Children's Healthcare of Atlanta, Egleston Hospital (CHOA). The goal was to evaluate whether manual anthropometry and 3D imaging performed consistently in a high-resource setting with adequate lighting and internet. The same training, detailed above, was used, and pathology staff notified the anthropometrists upon arrival of a case at the morgue. Manual anthropometry was to be performed prior to the start of the diagnostic autopsy. Significant challenges arose during data collection, including identification of eligible cases and timing to conduct anthropometry before the start of the diagnostic autopsy. Despite best efforts to coordinate between the study team and CHOA team, the study resulted in a limited sample size of 3 cases; thus, our results will focus on the Kenya site. .

Results

Sample characteristics are summarized in **Table 1**. There were no significant differences in sample characteristics between the pre- and post- training groups. The majority of children were under 2 years of age and were evenly distributed by sex. Proportions of stunting, wasting, and underweight were high, with a higher prevalence of stunting noted in the post-training group.

Evaluation of Quality- Digit Preference

In **Table 2**, prior to training, there was a clear tendency to round to the nearest 0.0 or 0.5 decimals for length, HC, and MUAC. There were no obvious signs of digit preference for weight measurement. The distribution of terminal digits post-training was evenly distributed for all measures. Similar patterns exist when examining the DPS. The DPS for length, HC and MUAC prior to the training exceeded the acceptable limit, while the DPS post-training were below the acceptable cutoff of 20.

Evaluation of Quality- Means and Standard Deviations of Anthropometric Indices

Table 3 summarizes the means and standard deviations for length-for-age (LAZ), weight-for-age (WAZ), and weight-for-length (WLZ), expressed as z scores. The standard deviations of all indices exceeded acceptable values both pre- and post-training. There were no differences in WAZ and WLZ pre- and post-training, but there was a statistically significant increase in LAZ post-training. There was a substantial loss in sample size when examining WLZ using WHO growth standards with 12% data loss in the pre- and 22% loss in the post-training group. There were no significant changes between the SDs for LAZ and WAZ pre- and post-training overall, and when stratified by age (<1 month vs 1-59 months as well as <6 months vs 6-59 months).

Evaluation of Precision-Technical Errors of measurement

Table 4 presents the TEMs and rTEMs specific to the post-training measures. The TEMs for length, weight, HC, and MUAC were, 0.32, 0.01, 0.18, and 0.13 respectively. The rTEMs for length, weight, HC, and MUAC were 0.53%, 0.29%, 0.48%, and 1.24%, respectively. All TEMs and rTEMs were within the acceptable range.

Accuracy- Spearman Correlation and Bland Altman Plots

Spearman correlation coefficients (Fig 1) comparing the manual measures to the 3D scans for length, MUAC, and HC were 0.99, 0.91, and 0.93, respectively. While the manual measures were highly correlated with the scans, the mean differences between scans and manual measures for length, MUAC, and HC were 1.61 cm, -0.20 cm, and 2.27 cm, respectively. These results suggest that the scans overestimate length by 1.61 cm, underestimate MUAC by 0.20 cm, and overestimate HC by 2.27 cm.

While there were challenges in securing data at the CHOA site, findings were complementary to those in the Kenya site. Among the 3 cases, standard anthropometry measurements were feasible and showed high precision (rTEMs for manual length, MUAC, and HC were 0.62%, 0.96%, and 1.80% respectively). For 3D scans, precision for duplicate scans was within acceptable limits when measuring length (rTEM=1.05%), but the software had more difficulty capturing precise measurements for MUAC (rTEM=4.71%) and HC (rTEM=1.62%).

Qualitative findings

The post-intervention survey revealed that all participants felt that training in manual anthropometry improved the accuracy of their measurements. Additionally, all participants reported feeling confident in their ability to perform manual anthropometry. While most participants (66.7%) believed that 3D imaging reduced measurement time in comparison to manual anthropometry, all participants overall preferred the use of manual anthropometry.

The qualitative findings from the in-depth interviews revealed that the team had a clear preference for manual anthropometry over the 3D imaging software as they felt the 3D imaging software required more time, better lighting, improved morgue environment, and training to ensure an accurate scan.

> *"We would take manual anthropometric measurements more seriously and would choose it well over 3D scanning...A lot of movement and manipulation of the camera to capture the entire body. And many times for 3D imaging, you have to repeat the process over and over and over again for you to be able to get the entire body into the screen. So it takes quite a bit more time…The boards work really well for us. It's a stable board… it's something we opt for over any other methods."*

Additionally, study investigators cited challenges in using the software when lighting was insufficient or when morgue environments varied.

> *"For what we experienced on the 3D, we had a few issues … our autopsy table had a fixed length and was not adjustable, so it was hard to get the complete image as you scan. Many times, we had issues with lighting systems. This made us end up with cut images—images with some parts of the body missing. So that called for checking and re-checking of images for quite a long period of time."*

Lastly, study investigators noted postmortem-specific challenges to manual anthropometry and understood the implications of taking careful measurement and attention to details to ensure data quality and minimize measurement.

> *"With rigor mortis, you will find that children stiffening, even the legs stiffening in some specific direction. If you are not able to manipulate them properly, one will end up with increased length as opposed to getting the accurate length. So that also required a lot of keenness."*

> *"The challenge in checking MUAC with tape measure comes when the subject you are measuring has reduced skin turgor. That is the skin of the arm becomes floppy. So that one might give you a lesser MUAC."*

Discussion

Following training on manual anthropometry and use of standard equipment for post-mortem assessment of nutritional status, data quality and precision improved; however, standard deviations of anthropometric indices pre- and post-training exceeded acceptable values. 3D imaging scans overestimated length by approximately 1.6 cm, underestimated MUAC by 0.2 cm, and overestimated HC by 2.3 cm. The presence of rigor mortis did not impede the

collection or quality of manual anthropometry measurements; however, additional care and pressure are critical to ensuring high quality data.

Digit preference improved for length, HC and MUAC following the training. There was no evidence of digit preference for weight pre- or post-training, which is likely due to how the measurements were taken. Weight was read from a digital scale, while length and circumference measurements were reliant on the anthropometrist's ability to use the equipment properly and read a tape measure accurately. Previous studies among living children have shown that the SD of anthropometric z-scores are reasonably consistent across populations, irrespective of nutritional status, and thus can be used to assess the quality of anthropometric data [32].The SD for all anthropometric indices exceeded acceptable limits both pre- and post-training, and sensitivity analyses revealed that high SDs for LAZ and WAZ were unlikely to be explained by age. If we continue with the conclusion that the intervention may have improved data quality and precision, then the persistently high SDs may be explained by capturing anthropometric measurements of small, severely ill children.

We also noted a decrease in sample size when examining WLZ scores. This is because nearly one-fourth of children in this sample fell below 45 cm, or the smallest length captured by the WHO growth standards when calculating WLZ [31]. The WHO growth standards were based on a healthy population of children, receiving optimal nutrition, raised in optimal environments, and receiving optimal healthcare - unlike the cases captured in CHAMPS. Many of the CHAMPS cases, at the end of life, had severe malnutrition and had body sizes not compatible with postnatal life and survival based on their chronologic age. Future research might consider application of the INTERGROWTH-21 (IG21-GS) standards [33] to classify nutritional status of children that fall outside of the WHO growth standards, such as in the case of severely ill cohorts of young children in CHAMPS.

This study has multiple strengths. First, to our knowledge, no research has been conducted on the feasibility of using gold-standard anthropometric assessment in the postmortem setting. Assessment of malnutrition and standardization of growth within the field of nutrition is typically based on z-scores derived from the 2006 WHO's Multicentre Growth Reference Study (MGRS). These standards are based on healthy, living children. Utilizing anthropometric data from CHAMPS, a large, multi-site surveillance system designed to elucidate the causes of U5M in high mortality regions of the world, may help inform the possible ranges of anthropometric deficits in severely ill populations. Second, our project captured staff reflections of conducting manual anthropometry of young children in field-based and clinical-morgue post-mortem settings. These qualitative findings may prove useful in informing strategies to improve the accuracy of post-mortem anthropometry.

This project was also subject to several limitations. First, in the CHOA site, we encountered unexpected obstacles in reaching our goal sample size due to limited time to perform the manual and 3D imaging anthropometric measurements before autopsies were performed. Further, the added data collection steps placed a significant burden on clinical staff and led to disruption of their workflow. Second, in Kenya, challenges arose with the 3D imaging software. The software settings were subject to user error and were altered during data collection, which

resulted in a compromised final sample size. Among the viable scans, our results suggest that the scans overestimated both length and HC. These findings are aligned with a recent study [24] and further suggest that before 3D imaging can be considered a viable, accurate alternative to manual anthropometry, adjustment of the technology and additional user testing is warranted to ensure reliable anthropometric measures.

Conclusions

Collection of quality anthropometric data following implementation of standardized training and equipment is feasible and reliable in postmortem field studies. While 3D imaging may be an accurate alternative to manual anthropometry, technology adjustments are needed to ensure accuracy and usability. Future research on the appropriate use of standards to define malnutrition among severely ill populations, including those in the post-mortem setting, are needed to elucidate our understanding of the role of malnutrition in U5M a.

Acknowledgements

The Child Health and Mortality Prevention Surveillance network would like to extend sincere appreciation to all the families who participated. Additionally, special thanks to Afrin Jahan for her analysis replication and figure development of Child Health and Mortality Prevention Surveillance (CHAMPS) network data related to this work.

AUTHOR CONTRIBUTIONS

PSS, PMG, KS, and JK designed and conducted the research. PMG analyzed the data, lead the manuscript development, and is primarily responsible for the final content. All authors have read and approved the final manuscript.

REFERENCES

1. *Children: improving survival and well-being*. 2020; Available from: https://www.who.int/news-room/fact-sheets/detail/children-reducing-mortality.
2. Pelletier, D.L., et al., *The effects of malnutrition on child mortality in developing countries.* Bull World Health Organ, 1995. **73**(4): p. 443-8.
3. Rice, A.L., et al., *Malnutrition as an underlying cause of childhood deaths associated with infectious diseases in developing countries.* Bull World Health Organ, 2000. **78**(10): p. 1207-21.
4. Schroeder, D.G. and K.H. Brown, *Nutritional status as a predictor of child survival: summarizing the association and quantifying its global impact.* Bull World Health Organ, 1994. **72**(4): p. 569-79.
5. Salzberg, N.T., et al., *Mortality Surveillance Methods to Identify and Characterize Deaths in Child Health and Mortality Prevention Surveillance Network Sites.* Clin Infect Dis, 2019. **69**(Suppl 4): p. S262-s273.
6. Suchdev, P.S., et al., *Assessment of Neurodevelopment, Nutrition, and Inflammation From Fetal Life to Adolescence in Low-Resource Settings.* Pediatrics, 2017. **139**(Suppl 1): p. S23-s37.
7. Cashin, K., Oot, L.,. *Guide to Anthropometry: A Practical Tool for Program Planners, Managers, and Implementers*. 2018; Available from: https://www.fantaproject.org/tools/anthropometry-guide.
8. Gupta, P.M., et al., *Improving assessment of child growth in a pediatric hospital setting.* BMC Pediatr, 2020. **20**(1): p. 419.
9. Wit, J.M., et al., *Practical Application of Linear Growth Measurements in Clinical Research in Low- and Middle-Income Countries.* Horm Res Paediatr, 2017. **88**(1): p. 79-90.
10. Conkle, J., et al., *Improving the quality of child anthropometry: Manual anthropometry in the Body Imaging for Nutritional Assessment Study (BINA).* PLoS One, 2017. **12**(12): p. e0189332.
11. Mwangome M, B.J., *Measuring infants aged below 6 months: experience from the field.* Field Exchange, 2014(47).
12. Bilukha, O., et al., *Comparison of anthropometric data quality in children aged 6-23 and 24-59 months: lessons from population-representative surveys from humanitarian settings.* BMC Nutrition, 2020. **6**(1).
13. Sachdeva, S., et al., *Mid-upper arm circumference v. weight-for-height Z-score for predicting mortality in hospitalized children under 5 years of age.* Public Health Nutr, 2016. **19**(14): p. 2513-20.
14. McCormack, C.A., et al., *Reliability of body size measurements obtained at autopsy: impact on the pathologic assessment of the heart.* Forensic Sci Med Pathol, 2016. **12**(2): p. 139-45.
15. Raghunathan, P.L., S.A. Madhi, and R.F. Breiman, *Illuminating Child Mortality: Discovering Why Children Die.* Clin Infect Dis, 2019. **69**(Suppl 4): p. S257-s259.
16. Blau, D.M., et al., *Overview and Development of the Child Health and Mortality Prevention Surveillance Determination of Cause of Death (DeCoDe) Process and DeCoDe Diagnosis Standards.* Clin Infect Dis, 2019. **69**(Suppl 4): p. S333-s341.
17. Mehta, A., *Abstract: Prevalence of undernutrition among children 1-59 months in the Child Health and Mortality Prevention Surveillance (CHAMPS) Network*, in *The 22nd International Union of Nutritional Sciences (IUNS)-International Congress of Nutrition (ICN)*. 2022: Tokyo, Japan.
18. Perumal, N., et al., *Anthropometric data quality assessment in multisurvey studies of child growth.* Am J Clin Nutr, 2020. **112**(Suppl 2): p. 806s-815s.

19. Corsi, D.J., J.M. Perkins, and S.V. Subramanian, *Child anthropometry data quality from Demographic and Health Surveys, Multiple Indicator Cluster Surveys, and National Nutrition Surveys in the West Central Africa region: are we comparing apples and oranges?* Glob Health Action, 2017. **10**(1): p. 1328185.
20. Prudhon, C., et al., *An algorithm to assess methodological quality of nutrition and mortality cross-sectional surveys: development and application to surveys conducted in Darfur, Sudan.* Popul Health Metr, 2011. **9**(1): p. 57.
21. Rakislova, N., et al., *Standardization of Minimally Invasive Tissue Sampling Specimen Collection and Pathology Training for the Child Health and Mortality Prevention Surveillance Network.* Clinical Infectious Diseases, 2019. **69**(Supplement_4): p. S302-S310.
22. Centers for Disease Control and Prevention, W.H.O., Nutrition International, UNICEF,. *Micronutrient survey manual*. 2020; Licence: CC BY-NC SA 3.0 IGO:[Available from: https://mnsurvey.nutritionintl.org/.
23. Jefferds, M.E.D., et al., *Acceptability and Experiences with the Use of 3D Scans to Measure Anthropometry of Young Children in Surveys and Surveillance Systems from the Perspective of Field Teams and Caregivers.* Current Developments in Nutrition, 2022. **6**(6).
24. Bougma, K., et al., *Accuracy of a handheld 3D imaging system for child anthropometric measurements in population-based household surveys and surveillance platforms: an effectiveness validation study in Guatemala, Kenya, and China.* The American Journal of Clinical Nutrition, 2022. **116**(1): p. 97-110.
25. Oliveira, T., et al., *Technical error of measurement in anthropometry (English version).* Revista Brasileira de Medicina do Esporte, 2005. **11**: p. 81-85.
26. SMART Action Against Hunger—Canada and Technical Advisory Group, *The SMART Plausibility Check for Anthropometry.* 2015.
27. Conkle, J., et al., *Improving the quality of child anthropometry: Manual anthropometry in the Body Imaging for Nutritional Assessment Study (BINA).* PloS one, 2017. **12**(12): p. e0189332-e0189332.
28. Mei, Z. and L.M. Grummer-Strawn, *Standard deviation of anthropometric Z-scores as a data quality assessment tool using the 2006 WHO growth standards: a cross country analysis.* Bull World Health Organ, 2007. **85**(6): p. 441-8.
29. Dirk Schumacher, *anthro: Computation of the WHO Child Growth Standards*. 2021.
30. RStudioTeam., *RStudio: Integrated Development Environment for R. RStudio, PBC,*. 2022: Boston, MA URL.
31. *WHO Child Growth Standards based on length/height, weight and age.* Acta Paediatr Suppl, 2006. **450**: p. 76-85.
32. Mei, Z. and L.M. Grummer-Strawn, *Standard deviation of anthropometric Z-scores as a data quality assessment tool using the 2006 WHO growth standards: a cross country analysis.* Bulletin of the World Health Organization, 2007. **85**(6): p. 441-448.
33. Papageorghiou, A.T., et al., *The INTERGROWTH-21(st) fetal growth standards: toward the global integration of pregnancy and pediatric care.* Am J Obstet Gynecol, 2018. **218**(2s): p. S630-s640.
34. Ulijaszek, S.J. and D.A. Kerr, *Anthropometric measurement error and the assessment of nutritional status.* Br J Nutr, 1999. **82**(3): p. 165-77.

TABLES

Table 1. Sample characteristics among pre- and post-intervention groups, Manyatta, Kenya

	Pre-intervention, n=75	Post-intervention, n=76	p-value[4]
**Age category, n (%)**			
<1 day	15 (20.0)	21 (27.6)	0.4821
1 day – 5 months	28 (37.3)	20 (26.3)	
6 – 23 months	23 (30.7)	25 (32.89)	
24 – 59 months	9 (12.0)	10 (13.1)	
**Sex, n (%)**			
Female	31 (41.3)	35 (46.1)	0.5589
**Anthropometric measurements, mean (SD)**			
Weight, kg	5.0 (3.8)	4.8 (3.5)	0.7543
Length, cm	62.0 (18.0)	60.0 (17.6)	0.4899
Head circumference (HC), cm	39.0 (6.9)	37.9 (7.4)	0.3509
Mid-Upper Arm Circumference (MUAC), cm	11.0 (3.0)	10.2 (3.0)	0.1064
**Nutritional status, n (%)**			
Stunting (LAZ[1]<-2SD)	24 (32.0)	38 (50.0)	0.0246
Wasting (WLZ[2]<-2SD)	58 (77.3)	54 (71.2)	0.3780
Underweight (WAZ[3]<-2)	40 (53.3)	50 (65.8)	0.1188

[1] LAZ: Length-for-age z-score
[2] WLZ: Length-for-weight z-score
[3] WAZ: Weight-for-age z-score
[4] p-values calculated using Chi Sq tests (age, sex, nutritional status) or t-tests (anthropometric measurements)

Table 2. Manual anthropometry digit preference scores[1] pre- and post-intervention, Manyatta, Kenya

	Pre- intervention, (N=75) n(%)				Post- intervention, (N=76) n(%)			
	**Length**	**Weight**	**HC**	**MUAC**	**Length**	**Weight**	**HC**	**MUAC**
**0.0**	65 (86.7)	15 (20.0)	57 (77.3)	54 (72.0)	3 (4.0)	10 (13.2)	5 (6.6)	2 (2.6)
**0.1**	-	2 (2.7)	-	-	12 (15.6)	8 (10.5)	11 (14.5)	18 (23.7)
**0.2**	-	6 (8.0)	-	-	7 (9.2)	9 (11.8)	9 (11.8)	10 (13.2)
**0.3**	-	9 (12.0)	-	-	13 (17.1)	4 (5.3)	3 (3.9)	9 (11.8)
**0.4**	-	6 (8.0)	-	-	5 (6.6)	9 (11.8)	9 (11.8)	5 (6.6)
**0.5**	10 (13.3)	6 (8.0)	17 (22.7)	21 (28.0)	6 (7.9)	9 (11.8)	8 (10.5)	9 (11.8)
**0.6**	-	11 (14.7)	-	-	7 (9.2)	10 (13.2)	13 (17.1)	5 (6.6)
**0.7**	-	9 (12.0)	-	-	8 (10.5)	4 (5.3)	1 (1.3)	5 (6.6)
**0.8**	-	5 (6.7)	-	-	8 (10.5)	6 (7.9)	12 (15.8)	8 (10.5)
**0.9**	-	6 (8.0)	-	-	7 (9.2)	7 (9.2)	5 (6.6)	5 (6.6)
**Digit preference score [1]**	86.2	15.3	78.1	74.3	10.4	9.5	16.6	18.4

[1] Digit preference scores computed using Mark Myatt and Ernest Guevarra (2022). nipnTK: National Information Platforms for Nutrition Anthropometric Data Toolkit. https://nutriverse.io/nipnTK/, https://github.com/nutriverse/nipnTK

DPS<20 is acceptable; ≥20 indicates digit preference is problematic

Table 3. Means and standard deviations for manual anthropometric indices, Manyatta, Kenya

	**Pre-training, (N=75)**		**Post-training, (N=76)**		**p-value[1]**	**Expected SD for high data quality [28]**
	n	Mean (SD)	n	Mean (SD)		
**LAZ[2] overall**	75	-1.1 (2.6)	76	-2.5 (2.9)	0.0018	**1.1 – 1.3**
**< 1 months**	35	-0.8 (2.8)	31	-3.0 (3.2)		
**1-59 months**	40	-1.4 (2.3)	45	-2.2 (2.7)		
**WAZ[3] overall**	75	-2.6 (2.3)	76	-3.2 (2.4)	0.0962	**1.0 – 1.2**
**< 1 months**	35	-2.0 (2.2)	31	-2.9 (2.2)		
**1-59 m months**	40	-3.1 (2.3)	45	-3.5 (2.5)		
**WLZ[4] overall**	66	-3.1 (1.8)	59	-2.9 (2.2)	0.4777	**0.85 – 1.1**
**< 1 months**	28	-2.6 (1.1)	15	-1.5 (1.3)		
**1-59 months**	38	-3.5 (2.1)	44	-3.3 (2.3)		

[1] p-values comparing overall pre- and post-training mean z-scores calculated using t-tests
[2] LAZ: Length-for-age z-score
[3] WLZ: Length-for-weight z-score
[4] WAZ: Weight-for-age z-score

Table 4. Manual anthropometry technical errors of measurement for post-intervention measures, Manyatta, Kenya,

	Length (cm)	Weight (kg)	Mid-Upper Arm Circumference (cm)	Head Circumference (cm)
**TEM[A]**	0.32	0.01	0.13	0.18
**Acceptable TEM [34]**	0.35	0.17	0.26	-
**VAV**	60.00	4.84	10.22	37.88
**Relative TEM (% TEM)[c]**	0.53%	0.29%	1.24%	0.48%

The technical error of measurement (TEM) is defined as the standard deviation of differences between repeated measures in the unit of the measurement, using the following equation

[A] Equation 1: absolute technical errors of measurement (TEM) = $\sqrt{\frac{\Sigma {d_i}^2}{2n}}$

Where:

$\Sigma {d_i}^2$ = Squared summation of deviations, n = number of individuals measured, and i = number of deviations

[C] Equation 2: relative TEM =$100\ x\ \frac{TEM}{VAV}$

Where TEM = technical error of measurement expressed as %, VAV= variable average value, the relative TEM (%TEM), and the coefficient of reliability (R) were the statistical tests used to assess intra- and inter-observer reliability. The TEM was defined as the standard deviation of differences between repeated measures in the unit of the measurement (e.g., TEM for height measured in centimeters is cm), using the following equation:

Skillful anthropometrists relative technical errors of measurement (%TEM) cutoff $\leq 1.5\%$
[25]

FIGURES

Figure 1. Bland Altman Plots for Length, Arm Circumference, and Head Circumference comparing manual anthropometry and 3D imaging, Manyatta, Kenya

Y-axis: the difference between the scans and the manual measurements by the average of the two methods

X-axis: the average of the scan and manual measures.

Dotted lines: represent the mean difference ± 3 standard deviations

Dashed lines: represent the mean difference ± 2 SD.

Solid line: across the plot is the no difference line.

Black points on the chart represent the 23 cases for which we had viable 3D scan data. Spearman correlation coefficients were examined to measure the strength of the relationship between scans and manual measures.

AC**:** Arm Circumference, HC**:** Head Circumference

Figure

Click here to access/download;Figure;Fig 1.tif

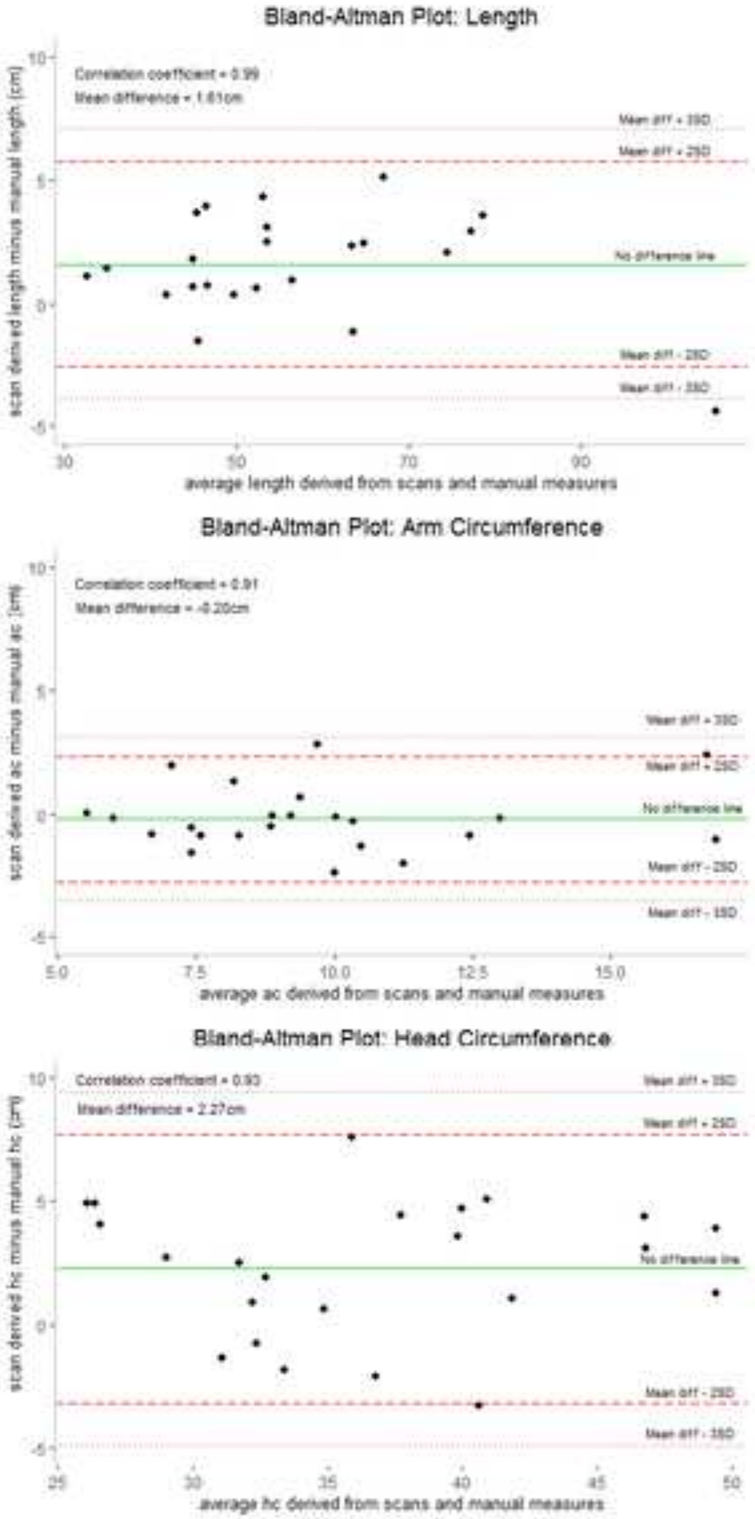

dataset2

Click here to access/download
Supporting Information
choa_anthropometry_evaluation_data.xlsx

dataset1

Click here to access/download

Supporting Information

kenya_anthropormetry_evaluation_data.xlsx

Impact of anthropometry training and feasibility of 3D imaging on anthropometry data quality among children under five years in a postmortem setting~~Feasibility and accuracy of performing manual anthropometry in the postmortem setting~~

Priya M. Gupta[1], Kasthuri Sivalogan[1], Richard Oliech[2], Eugene Alexander[3], Jamie Klein[4], O. Yaw. Addo[1,6], Dickson Gethi[5], Victor Akelo[5], Dianna M. Blau[6], Parminder S. Suchdev[1,~~2~~4,6*]

~~**Author affiliates**~~

1. Nutrition and Health Sciences Program, Laney Graduate School, Emory University, Atlanta, GA, USA
2. Kenya Medical Research Institute, Kisumu, Kenya
3. Body Surface Translations, Inc. (BST), Athens, GA, USA
4. Emory University School of Medicine, Atlanta, GA, USA
5. US Centers for Disease Control and Prevention-Kenya, Kisumu and Nairobi, Kenya
6. US Centers for Disease Control and Prevention, Atlanta, GA, USA

~~**Sources of Support:** This work was funded by grant OPP1126780 from the Bill & Melinda Gates Foundation~~

Conflicts of interest:
Eugene Alexander holds an ownership position in Body Surface Translations and therefore has a financial interest in the success of the 3D testing device described in this study. Data were blinded and not shared with Mr. Alexander until completion of draft manuscript.

Additional disclosure: The findings and conclusions in this report are those of the authors and do not necessarily represent the official position of the Centers for Disease Control and Prevention.

~~**Role of the Funder/Sponsor:** The funder participated in discussions of study design and data collection. They did not participate in the conduct of the study; the management, analysis, or interpretation of the data; preparation, review, or approval of the manuscript; or decision to submit the manuscript for publication.~~

Data Sharing: Data described in the manuscript, code book, and analytic code will be made available upon request pending [e.g., application and approval, payment, other].

***Corresponding Author** ~~**Information**~~
~~Priya M. Gupta~~
~~1534 Clifton Road~~
~~R-620~~
~~Atlanta, GA 30322~~
~~phone: 908-418-0970~~
~~pmgupta@emory.edu~~Parminder S Suchdev
1599 Clifton Rd NE

Atlanta, GA 30322
psuchde@emory.edu

Running Title: Anthropometry in the postmortem setting

ABSTRACT Abstract

BACKGROUND: The Child Health and Mortality Prevention Surveillance Network (CHAMPS) identifies causes of under-5 mortality in high mortality countries.

OBJECTIVE: To address challenges in postmortem nutritional assessment, we evaluated the impact of anthropometry training and the feasibility of 3D imaging on data quality within the CHAMPS Kenya site.

DESIGN: Staff were trained using World Health Organization (WHO)-recommended manual anthropometry equipment and novel 3D imaging methods to collect postmortem measurements. Following training, 76 deceased children were measured in duplicate and were compared to measurements of 75 pre-training deceased children. Outcomes included measures of data quality (standard deviations (SD) of anthropometric indices and digit preference scores (DPS)), precision (absolute and relative technical errors of measurement, TEMs or rTEMs), and accuracy (Bland-Altman plots). WHO growth standards (WHO GS) were used to produce anthropometric indices. Post-training surveys and in-depth interviews collected qualitative feedback on measurer experience with performing manual anthropometry and ease of using 3D imaging software.

RESULTS: Manual anthropometry data quality improved after training, as indicated by DPS. Standard deviations of anthropometric indices exceeded limits for high data quality when using the WHO growth standards GS. Reliability of measurements post-training was high as indicated by rTEMs below 1.5%. 3D imaging was highly correlated with manual measurements; however, on average 3D scans overestimated length and HC head circumference by 1.61 cm and 2.27 cm, respectively. Site staff preferred manual anthropometry to 3D imaging, as the imaging technology required adequate lighting and additional nuance considerations when performing the measurements.

CONCLUSIONS: Manual anthropometry was feasible and reliable postmortem in the presence of rigor mortis, and training improved digit preference. 3D imaging may be an accurate alternative to manual anthropometry, but technology adjustments are needed to ensure accuracy and usability. Future research on the appropriate use of current growth standards to define malnutrition in this severely ill population is needed.

Formatted: Font: 18 pt

Formatted: Font: 18 pt

Introduction~~INTRODUCTION~~

Formatted: Font: 18 pt

Malnutrition is estimated to contribute to approximately half of under-5-mortality (U5M) [1-3]. Malnutrition is also a major cause of morbidity as malnutrition plays a critical role in child neurodevelopment and health across the life course [2-4]. Reliable assessment tools for malnutrition are essential to reflect individual status, measure biological function, and predict health outcomes [5-7]. In children, inadequate growth is defined according to anthropometric measurements (length, weight, head and mid-upper arm circumference) that fall below 2 standard deviations of the normal sex-specific weight-for-length (wasting), length-for-age (stunting), and weight-for-age (underweight) [7]. Despite the importance of accurate anthropometry to detect early signs of malnutrition and monitor child growth, health facilities routinely use non-standardized anthropometric equipment, and as a result, measurements are often inaccurate [8]. Inaccurate measurements can lead to spurious classification of malnutrition in both individuals and populations[9].

In addition to the challenges of procuring and using standard anthropometric measurement tools, anthropometric measurements are subject to human error and are particularly difficult to collect among young children as children are easily distressed, have difficulty staying still, and may be unable to meet the requirements (i.e. ability to lie down or stand up) for manual anthropometry [10-12]. Anthropometric measurements are particularly challenging in hospitalized settings or in medically complex patients ~~due to difficulty taking measurements~~ due to medical equipment that may impede taking measurements (e.g., intravenous lines or ~~IV's,~~ feeding tubes), severe illness, or limitations in mobility. These children are also at highest risk of malnutrition [8, 13]. Additionally, qualitative findings from a quality improvement study in a children's hospital found that, wooden height-length measuring boards (ShorrBoard®, Weigh and Measure, LLC, Maryland USA) were considered to be "*heavy, cumbersome to assemble, frightening to patients, and required pre-planning and coordination between clinical staff with busy schedules and competing priorities*" [8]. Lastly, in field settings, the weight of the board may impede transportation and movement within the field and lack of standardization and maintenance of anthropometric equipment across study sites may contribute to poor data quality and misclassification [10, 11]. The post-mortem setting is another environment in which manual anthropometry may be challenging. Morgue capacity, rigor mortis, and edema can impact the quality and accuracy of measurements [14]. To our knowledge, no research has been conducted on the feasibility of using gold-standard anthropometric assessment in the postmortem setting.

The Child Health and Mortality Prevention Surveillance (CHAMPS) network is a multi-site surveillance system which strives to identify and understand the causes of under-5-mortality (U5M) in seven surveillance sites in sub-Saharan Africa and South Asia through detailed cause of death attribution with the use of high-quality postmortem anthropometrics, tissue samples, clinical abstraction, verbal autopsy, and the ability to integrate data from site-specific health and demographic surveillance systems (HDSS) [15, 16]. A recent analysis of the postmortem anthropometric data in CHAMPS suggested that nearly 90% of cases 1-59 months had evidence of undernutrition (stunting, wasting, or underweight) [17]. Given these data, it is possible that

malnutrition is directly or indirectly associated with child mortality. However, our understanding of the relationship between malnutrition and mortality may also be hindered by poor anthropometric measurement data quality, including digit preference (e.g. measurement rounding), high percentage of biologically implausible values, and standard deviations for anthropometric indices that exceed acceptable limits, which may lead to misclassification of malnutrition [18-20]. These data quality and precision outcomes may be a result of shortages of standard equipment in CHAMPS sites, lack of training on manual anthropometry, or difficulty in conducting manual anthropometry in the postmortem setting (rigor mortis, poor lighting in morgue facilities).

Our primary objectives were to determine whether manual anthropometry is feasible in the postmortem setting and to quantify the impact of training and standard equipment on data quality. Given the practical challenges of performing manual anthropometry in field and hospital-based settings, various 3D imaging approaches have also been developed to obtain anthropometric measurements. An efficacy study conducted at Emory University found that a 3D imaging software was as accurate as gold-standard manual anthropometry among under-5 children in Atlanta-area daycare centers [10]. However, data are also needed to assess 3D imaging in challenging hospital- or field-based settings. Therefore, our secondary objective was to assess the validity and acceptability of 3D imaging for anthropometric assessment compared to gold-standard manual anthropometry.

~~METHODS~~Materials and methods

Study site and data collection

This longitudinal quality improvement ~~anthropometry~~ study took place from October 2018 to September 2019 in the CHAMPS Manyatta, Kenya site located at the Jaramogi Oginga Odinga Teaching and Referral Hospital (JOOTRH). Prior to the training, site staff performed manual anthropometry on 75 deceased children as a routine part of the minimally invasive tissue sampling (MITS) portion of CHAMPS data collection. The MITS procedure is an abridged postmortem examination technique that has been validated for cause of death investigation in low-resource settings, described in detail ~~elsewhere~~ in an earlier study [21]. Written informed consent was obtained from families as part of the CHAMPS enrollment procedures. The CHAMPS protocol was approved by ethics committees in Kenya and at Emory University, Atlanta, GA, USA. Additional information regarding the ethical, cultural, and scientific considerations specific to inclusivity in global research is included in the Supporting Information.

Upon conclusion of pre-training data collection, a senior nutritionist, pediatrician, and anthropometry expert led and conducted an on-site 1-week training on manual anthropometry and the 3D imaging scanner for 6 staff. Using materials developed by the CDC, WHO and UNICEF, t~~T~~he training on manual anthropometry emphasized best practices for accurate manual measures of length, weight, ~~and~~ head circumference (HC) and mid-upper arm circumference (MUAC) measurements using two trained anthropometrists and standard

Formatted: Font: 18 pt

Formatted: Font: 16 pt, Bold

operating procedures [22]. Standard equipment in both sites, including wooden height-length measuring boards (ShorrBoard®, Weigh and Measure, LLC, Maryland USA), digital scales (Rice Lake Weighing Systems, Inc., Rice Lake, WI), and standard tape measures (Weigh and Measure LLC, Maryland USA), were used to ensure accurate measurement of recumbent length, weight, ~~and head circumference (~~HC~~)~~ and ~~mid-upper arm circumference (~~MUAC~~)~~, respectively. Staff completed an anthropometry standardization exercise using live children to ensure competence in conducting manual anthropometry. Staff were also trained on proper use the 3D imaging software using dolls and live children; details on the imaging software are provided ~~elsewhere~~ in earlier studies [10, 23, 24]. Briefly, the AutoAnthro system uses an iPad™ tablet, and a Structure Sensor™ camera attached to the tablet to capture non-personally identifiable anthropometric scan images of the deceased child. Following the training, two trained anthropometrists manually collected anthropometric measurements for 76 cases, with two separate measurements collected per case by different anthropometrists~~Following the training, two unique site staff each performed manual anthropometry on 76 new cases, for a total of 2 manual measures per case~~. Additionally, 3D scans were completed in duplicate for each anthropometrist, for a total of 4 scans per case. During data processing, after the completion of data collection, it was identified that the AutoAnthro software settings had been inadvertently altered for a significant number of cases, resulting in a final sample size of 23 cases. ~~Following data collection, it was found that the software settings had been inadvertently altered on the scanner resulting in viable scan data on only 23 cases~~.

Outcomes of interest

Key outcomes of interest included measures of data quality, precision, and accuracy. Data quality outcomes indicators included digit preference and standard deviations (SD) of anthropometric indices. ~~-~~Digit preference is the examination of a uniform distribution of terminal digits. We also calculated a digit preference score (DPS) to evaluate digit preference [25]. The DPS ranges from 0 to 100. Scores are low in instances of high agreement with the ideal of non-preference of the terminal digits, whereas DPS rises as the measures deviate from a uniform distribution across the terminal digits 0 through 9. In previous studies, a DPS cutoff above 20 was used to define the presence of digit preference [26, 27]. We thus used DPS<20 as acceptable, and DPS≥20 to indicate digit preference was problematic. Previous studies have suggested acceptable standard deviation ranges specifically for data quality among living children [28]. These include 1.10-1.30, 1.00-1.20, 0.85-1.10 for length-for-age (HAZ), weight-for-age (WAZ), and weight-for-length (WLZ) z-scores, respectively. Z-scores for anthropometric indices were produced using the ~~World Health Organization~~WHO Multicentre Growth Reference Study ~~growth standards (WHO-GS)~~ anthro R package [29].

Technical errors of measurement (TEM) were used to assess measurement precision. Following the training, the site staff performed manual anthropometry in duplicate. It is important to note that this differs from the data collection strategy pre-training in which a single set of measures were taken. As a result, we were only able to calculate TEMs for the data post-training in both sites. TEM express the error margin in anthropometry; they are unitless and allow comparison of errors across measures (e.g., weight, height etc.). Absolute TEMs were

Formatted: Font: 16 pt, Not Italic

calculated using the formula outlined in Equation 1 (**Table 4**). Absolute TEMs can also be transformed into relative TEMs, which express the error as a percentage corresponding to the total average. Relative TEMs (rTEM) were calculated using the formula outlined in Equation 2 (**Table 4**). We used a cutoff of <1.5% rTEM to indicate a skillful anthropometrist [25].

Finally, Bland Altman plots were used to assess the accuracy of the 3D imaging software relative to manual anthropometry following the training and were quantified in the unit of the measure (cm or kg). Spearman correlation coefficients examined the strength of the relationship between scans and manual measures.

Following the study, a short survey was sent to the 6 study participants. The survey collected information on whether the participants believed training on manual anthropometry improved the accuracy of the measurements, whether 3D imaging reduced the time to measure, and asked about the participants preference in measuring using manual anthropometry or the 3D imaging technology. We also conducted a 60-minute in-depth interview with the single lead site technician to collect qualitative feedback on the team's experience with performing manual anthropometry and ease of using the 3D imaging software. All analyses were conducted in ~~RStudio~~ R statistical software [30]. Statistical tests were two-sided and evaluated using an alpha level equal to 0.05. Pearson's Chi-Square tests (categorical variables) or t-tests (continuous variables) were used to evaluate differences between pre-intervention and post-interventions groups. The qualitative data were utilized to improve the implementation of manual anthropometric measurements across the CHAMPS Network.

We also conducted a small study in collaboration with the Pediatrics and Pathology departments at Children's Healthcare of Atlanta, Egleston Hospital (CHOA). The goal was to evaluate whether manual anthropometry and 3D imaging performed consistently in a high-resource setting with adequate lighting and internet. The same training, detailed above, was used, and pathology staff notified the anthropometrists upon arrival of a case at the morgue. Manual anthropometry was to be performed prior to the start of the diagnostic autopsy. Significant challenges arose during data collection, including identification of eligible cases and ~~availability~~ timing to conduct ~~manual~~ anthropometry before the start of the diagnostic autopsy. Despite best efforts to coordinate between the study team and CHOA team, the study resulted in a limited sample size of 3 cases; thus, our results will focus on the Kenya site. ~~Significant challenges arose during data collection which resulted in a limited sample size of 3 cases; thus, our results will focus on the Kenya site~~.

~~RESULTS~~Results

Formatted: Font: 18 pt

Sample characteristics are summarized in **Table 1**. There were no significant differences in sample characteristics between the pre- and post- training groups. The majority of children were under 2 years of age, and were evenly distributed by sex. Proportions of stunting, wasting, and underweight were high, with a higher prevalence of stunting noted in the post-training group.

Evaluation of Quality- Digit Preference

In **Table 2**, prior to training, there was a clear tendency to round to the nearest 0.0 or 0.5 decimals for length, HC, and MUAC. There were no obvious signs of digit preference for weight measurement. The distribution of terminal digits post-training was evenly distributed for all measures. Similar patterns exist when examining the DPS. The DPS for length, HC and MUAC prior to the training exceeded the acceptable limit, while the DPS post-training were ~~The DPS for length, pre-training, exceeded the acceptable limit, and post-training, the DPS for all measures fell~~ below the acceptable cutoff of 20.

Evaluation of Quality- Means and Standard Deviations of Anthropometric Indices

Table 3 summarizes the means and standard deviations for length-for-age (LAZ), weight-for-age (WAZ), and weight-for-length (WLZ), expressed as z scores. The standard deviations of all indices exceeded acceptable values both pre- and post-training. There were no differences in WAZ and WLZ pre- and post-training, but there was a statistically significant increase in LAZ post-training. There was a substantial loss in sample size when examining WLZ using WHO growth standards with 12% data loss in the pre- and 22% loss in the post-training group. ~~This decrease in sample size when examining WLZ scores is due to nearly a fourth of the children having lengths below 45 cm or the smallest length captured by the WHO growth standards when calculating WLZ [31]. It is important to note that the WHO growth standards were based on a healthy population of children, receiving optimal nutrition, raised in optimal environments, and receiving optimal healthcare – unlike the cases captured in CHAMPS. Many of the CHAMPS cases, at the end of life, had attained sizes that are more comparable to growth and nutritional status in utero and may explain why CHAMPS cases are not compatible with postnatal life and survival at their chronologic age.~~ There were no significant changes between the SDs for LAZ and WAZ pre- and post-training overall, and when stratified by age (<1 month vs 1-59 months as well as <6 months vs 6-59 months~~, data not shown~~).

Evaluation of Precision-Technical Errors of measurement

Table 4 presents the TEMs and rTEMs specific to the post-training measures.
The TEMs for length, weight, HC, and MUAC were, 0.32, 0.01, 0.18, and 0.13 respectively. The rTEMs for length, weight, HC, and MUAC were 0.53%, 0.29%, 0.48%, and 1.24%, respectively. All TEMs and rTEMs were within the acceptable range.

Accuracy- Spearman Correlation and Bland Altman Plots

Spearman correlation coefficients (Fig~~ure~~ 1) comparing the manual measures to the 3D scans for length, MUAC, and HC were 0.99, 0.91, and 0.93, respectively. While the manual measures were highly correlated with the scans, the mean differences between scans and manual measures for length, MUAC, and HC were 1.61 cm, -0.20 cm, and 2.27 cm, respectively. These results suggest that the scans overestimate length by 1.61 cm, underestimate MUAC by 0.20 cm, and overestimate HC by 2.27 cm.

Formatted: Font: 16 pt, Not Italic

Formatted: Font: 16 pt, Not Italic

Formatted: Font: 16 pt, Not Italic

Formatted: Font: 16 pt, Not Italic

Formatted: Font: Not Bold

While there were challenges in securing data at the CHOA site, findings were complementary to those in the Kenya site ~~(data not shown)~~. Among the 3 cases, standard anthropometry measurements were feasible and showed high precision (rTEMs for manual length, MUAC, and HC were 0.62%, 0.96%, and 1.80% respectively). For 3D scans, precision for duplicate scans was within acceptable limits when measuring length (rTEM=1.05%), but the software had more difficulty capturing precise measurements for MUAC (rTEM=4.71%) and HC (rTEM=1.62%).

Qualitative findings~~: Use of 3D imaging in morgue setting~~

Formatted: Font: 16 pt, Not Italic

The post-intervention survey revealed that all participants felt that training in manual anthropometry improved the accuracy of their measurements. Additionally, all participants reported feeling confident in their ability to perform manual anthropometry. While most participants (66.7%) believed that 3D imaging reduced measurement time in comparison to manual anthropometry, all participants overall preferred the use of manual anthropometry.

The qualitative findings from the in-depth interviews revealed that the team had a clear preference for manual anthropometry over the 3D imaging software as they felt the 3D imaging software required more time, ~~nuance (~~better lighting, ~~and~~ improved morgue environment ~~)~~, and training to ensure an accurate scan.

> *"We would take manual anthropometric measurements more seriously and would choose it well over 3D scanning...A lot of movement and manipulation of the camera to capture the entire body. And many times for 3D imaging, you have to repeat the process over and over and over again for you to be able to get the entire body into the screen. So it takes quite a bit more time…The boards work really well for us. It's a stable board… it's something we opt for over any other methods."*

Additionally, study investigators cited challenges in using the software when lighting was insufficient or when morgue environments varied.

> *"For what we experienced on the 3D, we had a few issues … our autopsy table had a fixed length and was not adjustable, so it was hard to get the complete image as you scan. Many times, we had issues with lighting systems. This made us end up with cut images—images with some parts of the body missing. So that called for checking and re-checking of images for quite a long period of time."*

Lastly, study investigators noted postmortem-specific challenges to manual anthropometry and understood the implications of taking careful measurement and attention to details to ensure ~~for~~ data quality and minimize measurement ~~error if careful measurement and attention to detail was not prioritized~~.

> *"With rigor mortis, you will find that children stiffening, even the legs stiffening in some specific direction. If you are not able to manipulate them properly, one will*

end up with increased length as opposed to getting the accurate length. So that also required a lot of keenness."

"~~T~~the challenge in checking MUAC with tape measure comes when the subject you are measuring has reduced skin turgor. That is the skin of the arm becomes floppy. So that one might give you a lesser MUAC."

~~DISCUSSION~~Discussion

Formatted: Font: 18 pt

Following training on manual anthropometry and use of standard equipment for post-mortem assessment of nutritional status, data quality and precision ~~were high~~improved;~~,~~ however, standard deviations of anthropometric indices pre- and post-training exceeded acceptable values. 3D imaging scans overestimated length by approximately 1.6 cm, underestimated MUAC by 0.2 cm, and overestimated HC by 2.3 cm. The presence of rigor mortis did not impede the collection or quality of manual- anthropometry ~~length~~ measurements; however, additional care and pressure are critical to ensuring high quality data.

Digit preference improved for length, HC and MUAC following the training. There was no evidence of digit preference for weight pre- or post-training, which is likely due to how the measurements were taken. Weight was read from a digital scale, while length and circumference measurements were reliant on the anthropometrist's ability to use the equipment properly and read a tape measure accurately. ~~Digit preference improved for length, HC and MUAC following the training.~~ Previous studies among living children have shown that the SD of anthropometric z-scores are reasonably consistent across populations, irrespective of nutritional status, and thus can be used to assess the quality of anthropometric data [32]. ~~In Kenya, t~~The SD for all anthropometric indices exceeded acceptable limits both pre- and post-training, and sensitivity analyses revealed that high SDs for LAZ and WAZ were unlikely to be explained by age. If we continue with the conclusion that the intervention may have ~~contributed to high~~improved data quality and precision, ~~then~~ then the persistently high SDs may be explained by capturing anthropometric measurements of small, severely ill children.

We also noted a decrease in sample size when examining WLZ scores. This is because nearly one-fourth of children in this sample fell below 45 cm, or the smallest length captured by the WHO growth standards when calculating WLZ [31]. ~~It is important to note that the~~The WHO growth standards were based on a healthy population of children, receiving optimal nutrition, raised in optimal environments, and receiving optimal healthcare - unlike the cases captured in CHAMPS. Many of the CHAMPS cases, at the end of life, had severe malnutrition and had body sizes ~~attained sizes that are more comparable to growth and nutritional status in utero and may explain why CHAMPS cases are~~ not compatible with postnatal life and survival ~~at~~ based on their chronologic age. Future research might consider application of the INTERGROWTH-21 (IG21-GS) standards [33] to classify nutritional status of children that fall outside of the WHO growth standards ~~GS~~, such as in the case of severely ill cohorts of young children ~~as~~ in CHAMPS ~~enrolled cases~~. ~~Comparing cases classified using the WHO-Gs versus IG21-GS would enables us to understand how these children would rank, had they had survived.~~

This study has multiple strengths. First, to our knowledge, no research has been conducted on the feasibility of using gold-standard anthropometric assessment in the postmortem setting. Assessment of malnutrition and standardization of growth within the field of nutrition is typically based on z-scores derived from the 2006 WHO's Multicentre Growth Reference Study (MGRS). These standards are based on healthy, living children~~, whereas being severely ill does not have a sufficient comparison group based on anthropometry~~. Utilizing anthropometric data from CHAMPS, ~~is~~ a large, multi-site surveillance system~~,~~ designed to elucidate the causes of U5M in high mortality regions of the world, ~~therefore these standardized anthropometric data~~ may help inform the possible ranges of anthropometric deficits in severely ill populations. Second, our project captured staff reflections ~~and criticisms~~ of conducting manual anthropometry of young children in field-based and clinical-morgue post-mortem settings~~in field-based and clinical-morgue settings~~. These qualitative findings may prove useful in informing strategies to improve the accuracy of~~te~~ post-mortem anthropometry~~ in field-based and clinical-morgue settings given the structural and practical constraints of the environment~~.

This project was also subject to several limitations. First, in the CHOA site, we encountered unexpected obstacles in reaching our goal sample size due to limited time to perform the manual and 3D imaging anthropometric measurements~~. We learned that not all deceased children undergo autopsy and not all cases are routed to the morgue via the pathology department. When cases were routed to the morgue, there was limited time to conduct standard anthropometry and 3D imaging in duplicate or~~ before autopsies were performed. Further, the added data collection steps ~~Second, the need for two anthropometrists to arrive at the morgue and collect data before autopsy~~ placed a significant burden on clinical staff and led to disruption of their workflow. ~~It should be noted, that within the CHOA site, autopsies are performed quickly and, in a step-wise fashion following the death of the child. There was often little time to balance case notification, standard equipment assembly and repeated measures. These challenges explain the limited sample size. Additionally, we found the pathologists were reluctant to using the 3D imaging software and the standard equipment. It appeared that knowledge of the importance of standard equipment was limited, although many had been introduced to the equipment earlier in their professional training. In the CHOA morgues, standard practice for securing postmortem measurements involved use of a tape measure, any deviations to this norm were resisted and were assumed to require additional time.~~ ~~Third~~Second, in Kenya, challenges arose with the 3D imaging software. The software settings were subject to user error and were altered during data collection, which resulted in a compromised final sample size. Among the viable scans, our results suggest that the scans overestimated both length and HC. These findings are aligned with a recent study [24] and further suggest that before 3D imaging can be considered a viable, accurate alternative to manual anthropometry, adjustment of the technology and additional user testing is warranted to ensure reliable anthropometric measures.

Conclusions

Formatted: Font: 18 pt, Bold

Collection of quality anthropometric data ~~and~~ following implementation of standardized training and equipment is feasible and reliable in ~~population-based,~~ postmortem~~,~~ field studies. While 3D imaging may be an accurate alternative to manual anthropometry, technology

adjustments are needed to ensure accuracy and usability. Future research on the appropriate use of standards to define malnutrition among severely ill populations, including those in the post-mortem setting, are needed to ~~will~~ elucidate our understanding of the role of malnutrition in U5M a~~nd inform future malnutrition-specific U5M reduction interventions~~.

~~ACKNOWLEDGEMENTS~~Acknowledgements

The Child Health and Mortality Prevention Surveillance network would like to extend sincere appreciation to all the families who participated. Additionally, special thanks to Afrin Jahan for her analysis replication and figure development of Child Health and Mortality Prevention Surveillance (CHAMPS) network data related to this work.

AUTHOR CONTRIBUTIONS

PSS, PMG, KS, and JK designed and conducted the research. PMG analyzed the data, lead the manuscript development, and is primarily responsible for the final content. All authors have read and approved the final manuscript.

Formatted: Font: 18 pt

REFERENCES

1. *Children: improving survival and well-being*. 2020; Available from: https://www.who.int/news-room/fact-sheets/detail/children-reducing-mortality.
2. Pelletier, D.L., et al., *The effects of malnutrition on child mortality in developing countries.* Bull World Health Organ, 1995. **73**(4): p. 443-8.
3. Rice, A.L., et al., *Malnutrition as an underlying cause of childhood deaths associated with infectious diseases in developing countries.* Bull World Health Organ, 2000. **78**(10): p. 1207-21.
4. Schroeder, D.G. and K.H. Brown, *Nutritional status as a predictor of child survival: summarizing the association and quantifying its global impact.* Bull World Health Organ, 1994. **72**(4): p. 569-79.
5. Salzberg, N.T., et al., *Mortality Surveillance Methods to Identify and Characterize Deaths in Child Health and Mortality Prevention Surveillance Network Sites.* Clin Infect Dis, 2019. **69**(Suppl 4): p. S262-s273.
6. Suchdev, P.S., et al., *Assessment of Neurodevelopment, Nutrition, and Inflammation From Fetal Life to Adolescence in Low-Resource Settings.* Pediatrics, 2017. **139**(Suppl 1): p. S23-s37.
7. Cashin, K., Oot, L.,. *Guide to Anthropometry: A Practical Tool for Program Planners, Managers, and Implementers*. 2018; Available from: https://www.fantaproject.org/tools/anthropometry-guide.
8. Gupta, P.M., et al., *Improving assessment of child growth in a pediatric hospital setting.* BMC Pediatr, 2020. **20**(1): p. 419.
9. Wit, J.M., et al., *Practical Application of Linear Growth Measurements in Clinical Research in Low- and Middle-Income Countries.* Horm Res Paediatr, 2017. **88**(1): p. 79-90.
10. Conkle, J., et al., *Improving the quality of child anthropometry: Manual anthropometry in the Body Imaging for Nutritional Assessment Study (BINA).* PLoS One, 2017. **12**(12): p. e0189332.
11. Mwangome M, B.J., *Measuring infants aged below 6 months: experience from the field.* Field Exchange, 2014(47).
12. Bilukha, O., et al., *Comparison of anthropometric data quality in children aged 6-23 and 24-59 months: lessons from population-representative surveys from humanitarian settings.* BMC Nutrition, 2020. **6**(1).
13. Sachdeva, S., et al., *Mid-upper arm circumference v. weight-for-height Z-score for predicting mortality in hospitalized children under 5 years of age.* Public Health Nutr, 2016. **19**(14): p. 2513-20.
14. McCormack, C.A., et al., *Reliability of body size measurements obtained at autopsy: impact on the pathologic assessment of the heart.* Forensic Sci Med Pathol, 2016. **12**(2): p. 139-45.
15. Raghunathan, P.L., S.A. Madhi, and R.F. Breiman, *Illuminating Child Mortality: Discovering Why Children Die.* Clin Infect Dis, 2019. **69**(Suppl 4): p. S257-s259.
16. Blau, D.M., et al., *Overview and Development of the Child Health and Mortality Prevention Surveillance Determination of Cause of Death (DeCoDe) Process and DeCoDe Diagnosis Standards.* Clin Infect Dis, 2019. **69**(Suppl 4): p. S333-s341.
17. Mehta, A., *Abstract: Prevalence of undernutrition among children 1-59 months in the Child Health and Mortality Prevention Surveillance (CHAMPS) Network*, in *The 22nd International Union of Nutritional Sciences (IUNS)-International Congress of Nutrition (ICN)*. 2022: Tokyo, Japan.
18. Perumal, N., et al., *Anthropometric data quality assessment in multisurvey studies of child growth.* Am J Clin Nutr, 2020. **112**(Suppl 2): p. 806s-815s.

19. Corsi, D.J., J.M. Perkins, and S.V. Subramanian, *Child anthropometry data quality from Demographic and Health Surveys, Multiple Indicator Cluster Surveys, and National Nutrition Surveys in the West Central Africa region: are we comparing apples and oranges?* Glob Health Action, 2017. **10**(1): p. 1328185.
20. Prudhon, C., et al., *An algorithm to assess methodological quality of nutrition and mortality cross-sectional surveys: development and application to surveys conducted in Darfur, Sudan.* Popul Health Metr, 2011. **9**(1): p. 57.
21. Rakislova, N., et al., *Standardization of Minimally Invasive Tissue Sampling Specimen Collection and Pathology Training for the Child Health and Mortality Prevention Surveillance Network.* Clinical Infectious Diseases, 2019. **69**(Supplement_4): p. S302-S310.
22. Centers for Disease Control and Prevention, W.H.O., Nutrition International, UNICEF,. *Micronutrient survey manual*. 2020; Licence: CC BY-NC SA 3.0 IGO:[Available from: https://mnsurvey.nutritionintl.org/.
23. Jefferds, M.E.D., et al., *Acceptability and Experiences with the Use of 3D Scans to Measure Anthropometry of Young Children in Surveys and Surveillance Systems from the Perspective of Field Teams and Caregivers.* Current Developments in Nutrition, 2022. **6**(6).
24. Bougma, K., et al., *Accuracy of a handheld 3D imaging system for child anthropometric measurements in population-based household surveys and surveillance platforms: an effectiveness validation study in Guatemala, Kenya, and China.* The American Journal of Clinical Nutrition, 2022. **116**(1): p. 97-110.
25. Oliveira, T., et al., *Technical error of measurement in anthropometry (English version).* Revista Brasileira de Medicina do Esporte, 2005. **11**: p. 81-85.
26. SMART Action Against Hunger—Canada and Technical Advisory Group, *The SMART Plausibility Check for Anthropometry.* 2015.
27. Conkle, J., et al., *Improving the quality of child anthropometry: Manual anthropometry in the Body Imaging for Nutritional Assessment Study (BINA).* PloS one, 2017. **12**(12): p. e0189332-e0189332.
28. Mei, Z. and L.M. Grummer-Strawn, *Standard deviation of anthropometric Z-scores as a data quality assessment tool using the 2006 WHO growth standards: a cross country analysis.* Bull World Health Organ, 2007. **85**(6): p. 441-8.
29. Dirk Schumacher, *anthro: Computation of the WHO Child Growth Standards*. 2021.
30. RStudioTeam., *RStudio: Integrated Development Environment for R. RStudio, PBC,*. 2022: Boston, MA URL.
31. *WHO Child Growth Standards based on length/height, weight and age.* Acta Paediatr Suppl, 2006. **450**: p. 76-85.
32. Mei, Z. and L.M. Grummer-Strawn, *Standard deviation of anthropometric Z-scores as a data quality assessment tool using the 2006 WHO growth standards: a cross country analysis.* Bulletin of the World Health Organization, 2007. **85**(6): p. 441-448.
33. Papageorghiou, A.T., et al., *The INTERGROWTH-21(st) fetal growth standards: toward the global integration of pregnancy and pediatric care.* Am J Obstet Gynecol, 2018. **218**(2s): p. S630-s640.
34. Ulijaszek, S.J. and D.A. Kerr, *Anthropometric measurement error and the assessment of nutritional status.* Br J Nutr, 1999. **82**(3): p. 165-77.

TABLES

Table 1. Sample characteristics among pre- and post-intervention groups,~~- CHAMPS Study,~~ Manyatta, Kenya~~, October 2018 to September 2019~~

	Pre-intervention, n=75	Post-intervention, n=76	p-value[4]
	~~n(%)~~		
**Age category, n (%)**			
<1 day	15 (20.0)	21 (27.6)	0.4821
1 day – 5 months	28 (37.3)	20 (26.3)	
6 – 23 months	23 (30.7)	25 (32.89)	
24 – 59 months	9 (12.0)	10 (13.1)	
**Sex, n (%)**			
Female	31 (41.3)	35 (46.1)	0.5589
**Anthropometric measurements, mean (SD)**			
Weight, kg	5.0 (3.8)	4.8 (3.5)	0.7543
Length, cm	62.0 (18.0)	60.0 (17.6)	0.4899
Head circumference (HC), cm	39.0 (6.9)	37.9 (7.4)	0.3509
Mid-Upper Arm Circumference (MUAC), cm	11.0 (3.0)	10.2 (3.0)	0.1064
**Nutritional status, n (%)**			
Stunting (LAZ[1]<-2SD)	24 (32.0)	38 (50.0)	0.0246
Wasting (WLZ[2]<-2SD)	58 (77.3)	54 (71.2)	0.3780
Underweight (WAZ[3]<-2)	40 (53.3)	50 (65.8)	0.1188

[1] LAZ: Length-for-age z-score
[2] WLZ: Length-for-weight z-score
[3] WAZ: Weight-for-age z-score
[4] p-values calculated using Chi Sq tests (age, sex, nutritional status) or t-tests (anthropometric measurements)

Table 2. Manual ~~A~~anthropometry d~~D~~igit preference scores[1] pre- and post-intervention, ~~CHAMPS Study,~~ Manyatta, Kenya~~, October 2018 to September 2019~~

	Pre- intervention, (N=75) n(%)				Post- intervention, (N=76) n(%)			
	**Length**	**Weight**	**HC**	**MUAC**	**Length**	**Weight**	**HC**	**MUAC**
**0.0**	65 (86.7)	15 (20.0)	57 (77.3)	54 (72.0)	3 (4.0)	10 (13.2)	5 (6.6)	2 (2.6)
**0.1**	-	2 (2.7)	-	-	12 (15.6)	8 (10.5)	11 (14.5)	18 (23.7)
**0.2**	-	6 (8.0)	-	-	7 (9.2)	9 (11.8)	9 (11.8)	10 (13.2)
**0.3**	-	9 (12.0)	-	-	13 (17.1)	4 (5.3)	3 (3.9)	9 (11.8)
**0.4**	-	6 (8.0)	-	-	5 (6.6)	9 (11.8)	9 (11.8)	5 (6.6)
**0.5**	10 (13.3)	6 (8.0)	17 (22.7)	21 (28.0)	6 (7.9)	9 (11.8)	8 (10.5)	9 (11.8)
**0.6**	-	11 (14.7)	-	-	7 (9.2)	10 (13.2)	13 (17.1)	5 (6.6)
**0.7**	-	9 (12.0)	-	-	8 (10.5)	4 (5.3)	1 (1.3)	5 (6.6)
**0.8**	-	5 (6.7)	-	-	8 (10.5)	6 (7.9)	12 (15.8)	8 (10.5)
**0.9**	-	6 (8.0)	-	-	7 (9.2)	7 (9.2)	5 (6.6)	5 (6.6)
**Digit preference score [1]**	86.2	15.3	78.1	74.3	10.4	9.5	16.6	18.4

[1] Digit preference scores ~~(DPS)~~ computed using Mark Myatt and Ernest Guevarra (2022).
~~[2]~~ nipnTK: National Information Platforms for Nutrition Anthropometric Data Toolkit. https://nutriverse.io/nipnTK/, https://github.com/nutriverse/nipnTK
DPS<20 is acceptable; ≥20 indicates digit preference is problematic

Table 3. Means and standard deviations for manual anthropometric indices, ~~CHAMPS Study,~~ Manyatta, Kenya~~, October 2018 to September 2019~~

	Pre-training, (N=75)		Post-training, (N=76)		p-value[1]	Expected SD for high data quality [28]
	n	Mean (SD)	n	Mean (SD)		
**LAZ[2] overall**	75	-1.1 (2.6)	76	-2.5 (2.9)	0.0018	**1.1 – 1.3**
**< 1 months**	35	-0.8 (2.8)	31	-3.0 (3.2)		
**1-59 months**	40	-1.4 (2.3)	45	-2.2 (2.7)		
**WAZ[3] overall**	75	-2.6 (2.3)	76	-3.2 (2.4)	0.0962	**1.0 – 1.2**
**< 1 months**	35	-2.0 (2.2)	31	-2.9 (2.2)		
**1-59 m months**	40	-3.1 (2.3)	45	-3.5 (2.5)		
**WLZ[4] overall**	66	-3.1 (1.8)	59	-2.9 (2.2)	0.4777	**0.85 – 1.1**
**< 1 months**	28	-2.6 (1.1)	15	-1.5 (1.3)		
**1-59 months**	38	-3.5 (2.1)	44	-3.3 (2.3)		

[1] p-values comparing overall pre- and post-training mean z-scores calculated using t-tests
[2] LAZ: Length-for-age z-score
[3] WLZ: Length-for-weight z-score
[4] WAZ: Weight-for-age z-score

Table 4. Manual anthropometry ~~T~~technical errors of measurement for post-intervention measures, ~~CHAMPS Study,~~ Manyatta, Kenya, ~~October 2018 to September 2019~~

	Length (cm)	Weight (kg)	Mid-Upper Arm Circumference (cm)	Head Circumference (cm)
**TEM[A]**	0.32	0.01	0.13	0.18
**Acceptable TEM [34]**	0.35	0.17	0.26	-
**VAV**	60.00	4.84	10.22	37.88
**Relative TEM (% TEM)[c]**	0.53%	0.29%	1.24%	0.48%

The technical error of measurement (TEM) is defined as the standard deviation of differences between repeated measures in the unit of the measurement, using the following equation

[A] Equation 1: absolute technical errors of measurement (TEM) = $\sqrt{\frac{\Sigma d_i^{\ 2}}{2n}}$

Where:

$\Sigma d_i^{\ 2}$ = Squared summation of deviations, n = number of individuals measured, and i = number of deviations

[C] Equation 2: relative TEM $= 100\ x\ \frac{TEM}{VAV}$

Where TEM = technical error of measurement expressed as %, VAV= variable average value, the relative TEM (%TEM), and the coefficient of reliability (R) were the statistical tests used to assess intra- and inter-observer reliability. The TEM was defined as the standard deviation of differences between repeated measures in the unit of the measurement (e.g., TEM for height measured in centimeters is cm), using the following equation:

Skillful anthropometrists relative technical errors of measurement (%TEM) cutoff $\leq 1.5\%$
[25]

FIGURES

Figure 1. Bland Altman Plots for Length, Arm Circumference, and Head Circumference comparing manual anthropometry and 3D imaging, ~~CHAMPS Study,~~ Manyatta, Kenya~~, October 2018 to September 2019~~

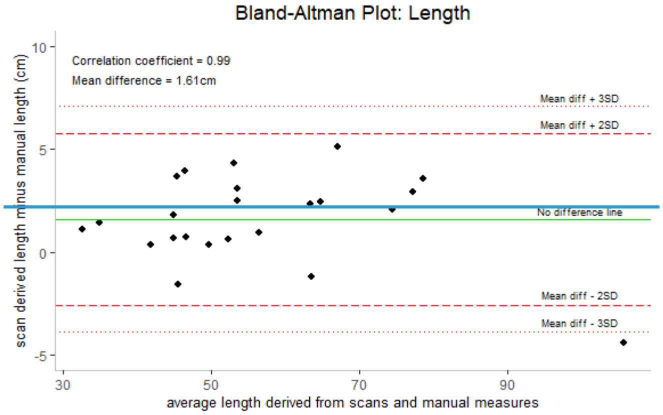

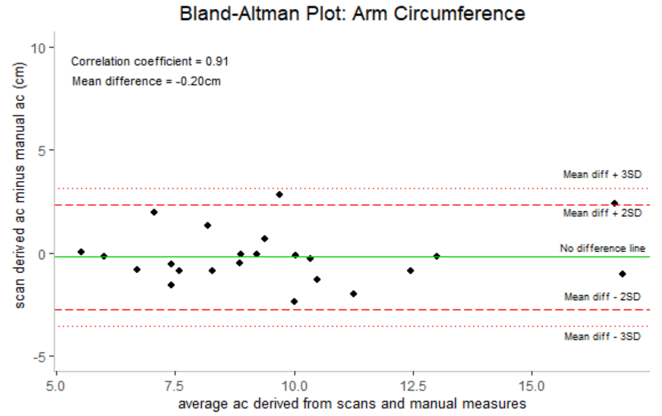

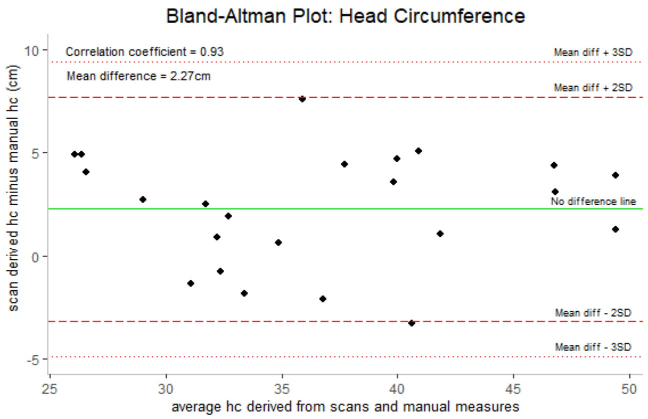

Y-axis: the difference between the scans and the manual measurements by the average of the two methods
X-axis: the average of the scan and manual measures.

Dotted lines: represent the mean difference ± 3 standard deviations
Dashed lines: represent the mean difference ± 2 SD.
Solid line: across the plot is the no difference line.

Black points on the chart represent the 23 cases for which we had viable 3D scan data. Spearman correlation coefficients were examined to measure the strength of the relationship between scans and manual measures.

AC**:** Arm Circumference, HC**:** Head Circumference

Formatted: Font: 12 pt

Response to Reviewers: PONE-D-23-01635

Thank you for the thoughtful and detailed review of our manuscript. We have carefully revised and responded to each point raised by the academic editor and reviewers noted below in red font. We have also submitted a revised manuscript in track changes, as well as an unmarked revised manuscript.

Editor comments

1. Please ensure that your manuscript meets PLOS ONE's style requirements, including those for file naming. The PLOS ONE style templates can be found at

https://journals.plos.org/plosone/s/file?id=wjVg/PLOSOne_formatting_sample_main_body.pdf and https://journals.plos.org/plosone/s/file?id=ba62/PLOSOne_formatting_sample_title_authors_affiliations.pdf

- Thanks we have made the required style changes including resubmitted the figure as a tiff file.

2. Please include a complete copy of PLOS' questionnaire on inclusivity in global research in your revised manuscript. Our policy for research in this area aims to improve transparency in the reporting of research performed outside of researchers' own country or community. The policy applies to researchers who have travelled to a different country to conduct research, research with Indigenous populations or their lands, and research on cultural artefacts. The questionnaire can also be requested at the journal's discretion for any other submissions, even if these conditions are not met.

Please find more information on the policy and a link to download a blank copy of the questionnaire here: https://journals.plos.org/plosone/s/best-practices-in-research-reporting.

Please upload a completed version of your questionnaire as Supporting Information when you resubmit your manuscript.

- Thanks we have completed and questionnaire and uploaded it as supporting information. We have also referred to the checklist in our Methods section

3.Please provide additional details regarding participant consent. In the ethics statement in the Methods and online submission information, please ensure that you have specified (1) whether consent was informed and (2) what type you obtained (for instance, written or verbal, and if verbal, how it was documented and witnessed). If your study included minors, state whether you obtained consent from parents or guardians. If the need for consent was waived by the ethics committee, please include this information.

If you are reporting a retrospective study of medical records or archived samples, please ensure that you have discussed whether all data were fully anonymized before you accessed them and/or whether the IRB or ethics committee waived the requirement for informed consent. If patients provided informed written consent to have data from their medical records used in research, please include this information.

- We have added 2 sentences in the Methods section describing both consent and study ethical clearance.

4. Thank you for stating the following in the Competing Interests section:

"I have read the journal's policy and the authors of this manuscript have the following competing interests: Eugene Alexander holds an ownership position in Body Surface Translations and therefore has a financial interest in the success of the 3D testing device described in this study. Data were blinded and not shared with Mr. Alexander until completion of draft manuscript.

Additional disclosure: The findings and conclusions in this report are those of the authors and do not necessarily represent the official position of the Centers for Disease Control and Prevention."

Please confirm that this does not alter your adherence to all PLOS ONE policies on sharing data and materials, by including the following statement: ""This does not alter our adherence to PLOS ONE policies on sharing data and materials.” (as detailed online in our guide for authors http://journals.plos.org/plosone/s/competing-interests).

If there are restrictions on sharing of data and/or materials, please state these. Please note that we cannot proceed with consideration of your article until this information has been declared.

Please include your updated Competing Interests statement in your cover letter; we will change the online submission form on your behalf.

- Thanks, we have updated our competing interests statement to include “This does not alter our adherence to PLOS ONE policies on sharing data and materials.”

5. We note that you have indicated that data from this study are available upon request. PLOS only allows data to be available upon request if there are legal or ethical restrictions on sharing data publicly. For more information on unacceptable data access restrictions, please see http://journals.plos.org/plosone/s/data-availability#loc-unacceptable-data-access-restrictions.

In your revised cover letter, please address the following prompts:

a) If there are ethical or legal restrictions on sharing a de-identified data set, please explain them in detail (e.g., data contain potentially sensitive information, data are owned by a third-party organization, etc.) and who has imposed them (e.g., an ethics committee). Please also provide contact information for a data access committee, ethics committee, or other institutional body to which data requests may be sent.

b) If there are no restrictions, please upload the minimal anonymized data set necessary to replicate your study findings as either Supporting Information files or to a stable, public repository and provide us with the relevant URLs, DOIs, or accession numbers. For a list of acceptable repositories, please see http://journals.plos.org/plosone/s/data-availability#loc-recommended-repositories.

We will update your Data Availability statement on your behalf to reflect the information you provide.

- We have decided to share an anonymized dataset as Supporting Information files

6. We note that you have stated that you will provide repository information for your data at acceptance. Should your manuscript be accepted for publication, we will hold it until you provide the relevant accession numbers or DOIs necessary to access your data. If you wish to make changes to your Data Availability statement, please describe these changes in your cover letter and we will update your Data Availability statement to reflect the information you provide.

-As noted above, we have included our analysis datasets as a Supporting information file.

7. We note that you have included the phrase “data not shown” in your manuscript. Unfortunately, this does not meet our data sharing requirements. PLOS does not permit references to inaccessible data. We require that authors provide all relevant data within the paper, Supporting Information files, or in an acceptable, public repository. Please add a citation to support this phrase or upload the data that corresponds with these findings to a stable repository (such as Figshare or Dryad) and provide and URLs, DOIs, or accession numbers that may be used to access these data. Or, if the data are not a core part of the research being presented in your study, we ask that you remove the phrase that refers to these data.

- We have removed these statements as the data used are being shared as supporting information files

8. Please include a separate caption for figure in your manuscript.

- Done

Reviewers' comments:

Reviewer #1: The study is a very interesting study that will provide informaiton on how the errors in manual anthropometry can be improved in a post-mortem setting. Information claimed to be evaluated in the study will be very vital in nutrition assessment of children in post-mortem settings. However, the following observations were made during review:

1. The title of the article seem not to be approriate. Suggested title is presented in the comment section.

- Thanks for this feedback. Based on your and reviewer #2’s feedback, we have changed titled to “Impact of anthropometry training and feasibility of 3D imaging on anthropometry data quality among children under five years in a postmortem setting”

2. comments on the abstract, introduction and materials and methods, and results were made in the manuscript.

- Thank you for your suggestions. We have made all the suggested changes to the abstract, introduction, and methods sections.

3. Generally, authors seem not to define the objectives of the study properly and this is reflecting in the methodology, and result sections.

- We apologize for the confusion. We have tried to clearly define the objectives in the last paragraph of the introduction with both primary and secondary objectives. The methods and results section follow this same flow (e.g., presenting results of standard anthropometry data quality first, followed by study on 3D imaging)

4. Authors should interprete what is on the table correctly in the result section.
- We have reviewed and double-checked that all results in the table match their descriptions in the text.

5. The authors need to state the objectives of the study clearly and present results based on these objectives.

- please see response #3

6. Statistical analysis carried out was not clearly stated in the methodology. It is not appropriate to have to look at the table before having an idea of the statistics carried out.
- We have added a description of the statistical analyses conducted in the methods section

7. Figures indicated on the manuscript were not seen.
- We apologize- the figure was uploaded as a word document. It has now been resubmitted using the required tiff format

8. In the methodology, authors claimed to do a survey to collect information on whether the participants believed training on manual anthropometry improved the accuracy of the measurements, whether 3D imaging reduced the time to measure, and asked about the participants preference in measuring using manual anthropometry or the 3D imaging technology. In addition, authors also claimed to conducted a 60-minute in-depth interview with the single lead site technician to collect qualitative feedback on the team's experience with performing manual anthropometry and ease of using the 3D imaging software. The results for these survey and qualitative study are not clear in the result sections as well as the tables.

Results were presented not indicating whether it is for manual anthropometry or 3D imaging (Tables 1-4). Although, I suppose that is for manual anthropometry. The results for pre- and post-training for the 3D scan were never presented and figure 1 was comparing manual anthropometry with 3 D imaging (although the figures were not seen).
Results on qualitative feedbacks were only presented for 3D imaging and not manual anthropometry.
- Thank you for this feedback. We have attempted to make the Methods and results section more clear. We have clarified table titles to indicate manual anthropometry, and have also changed the Figure title to clarify. We have also added additional text in the qualitative results section (lines 301-305).

9. Results on whether participants believed training on manual anthropometry improved the accuracy of the measurement or 3D imaging reduced the time to measure were not presented at all. Also,

participants preference in measuring using manual anthropometry or 3D imaging technology was not presented in the result section.
- Thank you for raising this issue. We inadvertently excluded results from the post-training survey and have now added this to the "Qualitative findings" section of the results section (lines 300-305).

10. Discussion section needs to be re-written to reflect exactly what is in the results. In addition, results need to be properly discussed in line with findings from previous studies and implications should be discussed clearly and appropriately. Assumptions in the result sections is not approriate.

- Thanks for your comment. We have attempted to re-write sections of the Discussion section to improve clarity and insure that findings are consistent to what were reported in the Results.

11.Authors did not have conclusion section at all.

- The last paragraph was our conclusions, which has now been appropriately labeled. The wording of the Conclusion has also been updated.

12. There is need for proper organization of the content of the manuscripts for coherence.
- Thank you for your comment. We have added several new headers and text to improve the organization and flow of the manuscript

13. Some sentences seem complicated and difficult to understand. The authors are advised to seek for professional English editing service to check the revised manuscript for grammar, syntax and style errors.

- Thanks for your comment. We have made appropriate grammatical and style edits to improve clarity.

Reviewer #2: PONE-D-23-01635
This is an important topic because the findings add to the existing evidence on causes of death due to anthropometric deficits in children. This study is novel and a useful contribution to the body of evidence on child health and nutrition. The paper fits the PlosOne journal's aim and will be interesting to your readership.

The analysis is comprehensive and accurate. Limitations and strengths of the analysis have been declared adequately. Data analysis and results were adequately done and well presented. This paper deserves to be published.

Please, find below suggested minor comments and suggestions for your consideration to further improve your manuscript:

Comments:
Title
1. The current title of the article should be modified to reflect manual anthropometry as well as 3D imaging and the target or study population - children under 5 years.

- Thank you for your feedback. We have changed title to "Impact of anthropometry training and feasibility of 3D imaging on anthropometry data quality among children under five years in a postmortem setting"

Abstract

1. Please, could the conclusion "Future research on the appropriate use of current growth standards to define malnutrition in this severely ill population is needed" be revised to reflect the topic of interest. …. This severely ill population is not very clear, I thought the study setting was post-mortem.

- Thank you. We have modified the conclusion in the abstract and removed the sentence on use of growth standards, as this requires additional explanation in the Discussion section of the manuscript.

Method

1. In paragraph 3 …. weight, and circumference measurements using two…. please could indicate which circumference measurement you are referring to?

- Thank you for your feedback. We have updated the text in line 162 to reflect that head and mid-upper arm circumference are the two circumference measurements.

2. Please revise the sentence in paragraph 3 …. 'Following the training, two unique site staff each performed manual anthropometry on 76 new cases, for a total of 2 manual measures per case' and make it simpler and clearer.

- Thank you for the feedback. We revised lines 174-154 to say "Following the training, two trained anthropometrists manually collected anthropometric measurements for 76 cases, with two separate measurements collected per case by different anthropometrists".

3. Authors should check if the sentence in paragraph 3 "Following data collection, it was found that the software… settings had been inadvertently altered on the scanner resulting in viable scan data on only 23 cases." is communicating the right message, because if the software was inadvertently altered then it could me the data was not viable. I may be wrong. If that was true, then how did it impact on the findings?

-Thank you for the feedback. We revised lines 177-178 to say "During data processing, after the completion of data collection, it was identified that the AutoAnthro software settings had been inadvertently altered for a significant number of cases, resulting in a final sample size of 23 cases".

4. Clarity needed what actually happened? ……'Manual anthropometry was to be performed prior to the start of the diagnostic autopsy. Significant challenges arose during data collection which resulted in a limited sample size of 3 cases; thus, our results will focus on the Kenya site'.

- Thank you for the feedback. We have clarified line 230 to say "Significant challenges arose during data collection, including identification of eligible cases and availability to conduct manual anthropometry before the start of the diagnostic autopsy. Despite best efforts to coordinate between the study team and CHAO team, the study resulted in a limited sample size of 3 cases; thus, our results will focus on the Kenya site.

5. What was the duration of the training? What was the duration of the data collection?

-The Kenya site training took place in April 2019 followed by data collection from April-September 2019. The training for the CHOA study took place in September 2019 with data collection in October-November 2019.

6. Please indicate how you analysed the qualitative data, and how you utilized the data.

-Thank you for the feedback. We added information on the statistical analysis of the qualitative data and how we utilized the qualitative data in lines 219-223.

Results

1. 'There was a substantial loss in sample size when examining WLZ using WHO growth standards with 12% data loss in the pre- and 22% loss in the post-training group'.
How was LAZ also affected given that there was data lost for WLZ?
- Thanks for this question. When using the WHO growth standards, the absolute limit of 45cm in length only applies to the calculation of WLZ, not LAZ since LAZ is estimable because it is based on standardizing child length according to their completed age (and by sex). 45cm was determined by WHO as the minimum birth length for healthy children (e.g., without any intrauterine growth restriction and or congenital disorder of size).

2. Why will authors talk about results that are not available?
'While there were challenges in securing data at the CHOA site, findings were complementary to those in the Kenya site (data not shown)'.
- In response to earlier comment by editor, we have deleted the statement "data not shown" and have shared datasets as supporting files.

Discussion

1. Authors, please explain why rigor mortis will not impede manual anthropometry measurements, this is because the qualitative findings show that it could be a challenge to get accurate measurements.

- Thanks for this comment. We have edited the qualitative findings in the results to make this more clear. While there were challenges in taking manual anthropometric measurements due to rigor mortis, the stiffening was always accounted for with added pressure and time. Thus, the accuracy of measurements was ensured using the wooden length boards.

2. Table 3 in the results section and Paragraph 3 in the discussion have some repetitions, this happened because you cited literature in your results section. Authors should consider to present only results under the results section.

-Thank you for the feedback. We removed the duplicated information from Table 3 in the results section.

3. Check WHO-GS should be written as WHO-MGRS.

-Thanks for this comment. We have written out WHO growth standards and WHO Multicentre Growth Reference Study when appropriate.

4. ‘Future research on the appropriate use of standards to define malnutrition among severely ill populations will elucidate our understanding of the role of malnutrition in U5M and inform future malnutrition-specific U5M reduction interventions’ I think I know what you are trying to say but I am wandering if this appropriate recommendation because you did not work with severely sick children. Please consider to revise your recommendation.

- Thanks for this comment. We have revised our concluding statement.

General comments

1. Please number the lines for the sentences in your manuscript. It helps reviewers to give feedback easily.

- Line numbers have been added
