# Supplementary material for: Impact of anthropometry training and feasibility of 3D imaging on anthropometry data quality among children under five years in a postmortem setting
Source: PLoS One. 2023 Sep 28;18(9):e0292046. doi: 10.1371/journal.pone.0292046 (PMC10538800; doi:10.1371/journal.pone.0292046)
Supplement: Attachment — Submitted filename: Gupta_Suchdev_Manuscript_Revised20JAN23.docx [file pone.0292046.s003.docx]

**Feasibility and accuracy of performing manual anthropometry in the postmortem setting**

Priya M. Gupta^1^, Kasthuri Sivalogan^1^, Richard Oliech^2^, Eugene Alexander^3^, Jamie Klein^4^, O. Yaw. Addo^1,6^, Dickson Gethi^5^, Victor Akelo^5^, Dianna M. Blau^6^, Parminder S. Suchdev^1,2,6^

**Author affiliates**

1. Nutrition and Health Sciences Program, Laney Graduate School, Emory University, Atlanta, GA, USA
2. Kenya Medical Research Institute, Kisumu, Kenya
3. Body Surface Translations, Inc. (BST), Athens, GA, USA
4. Emory University School of Medicine, Atlanta, GA, USA
5. US Centers for Disease Control and Prevention-Kenya, Kisumu and Nairobi, Kenya
6. US Centers for Disease Control and Prevention, Atlanta, GA, USA

**Sources of Support:** This work was funded by grant OPP1126780 from the Bill & Melinda Gates Foundation

**Conflicts of interest:**

Eugene Alexander holds an ownership position in Body Surface Translations and therefore has a financial interest in the success of the 3D testing device described in this study. Data were blinded and not shared with Mr. Alexander until completion of draft manuscript.

**Additional disclosure:** The findings and conclusions in this report are those of the authors and do not necessarily represent the official position of the Centers for Disease Control and Prevention.

**Role of the Funder/Sponsor:** The funder participated in discussions of study design and data collection. They did not participate in the conduct of the study; the management, analysis, or interpretation of the data; preparation, review, or approval of the manuscript; or decision to submit the manuscript for publication.

**Data Sharing:**	Data described in the manuscript, code book, and analytic code will be made available upon request pending [e.g., application and approval, payment, other].

**Corresponding Author Information**
Priya M. Gupta

1534 Clifton Road

R-620

Atlanta, GA 30322

phone: 908-418-0970

[pmgupta@emory.edu](mailto:pmgupta@emory.edu)

**Running Title:** Anthropometry in the postmortem setting

**ABSTRACT**

**BACKGROUND:** The Child Health and Mortality Prevention Surveillance Network (CHAMPS) identifies causes of under-5 mortality in high mortality countries.

**OBJECTIVE**: To address challenges in postmortem nutritional assessment, we evaluated the impact of anthropometry training and the feasibility of 3D imaging on data quality within the CHAMPS Kenya site.

**DESIGN:** Staff were trained using World Health Organization (WHO)-recommended manual anthropometry equipment and novel 3D imaging methods to collect postmortem measurements. Following training, 76 deceased children were measured in duplicate and were compared to measurements of 75 pre-training deceased children. Outcomes included measures of data quality (standard deviations (SD) of anthropometric indices and digit preference scores (DPS)), precision (absolute and relative technical errors of measurement, TEMs or rTEMs), and accuracy (Bland-Altman plots). WHO growth standards (WHO-GS) were used to produce anthropometric indices. Post-training surveys and in-depth interviews collected qualitative feedback on measurer experience with performing manual anthropometry and ease of using 3D imaging software.

**RESULTS:** Manual anthropometry data quality improved after training, as indicated by DPS. Standard deviations of anthropometric indices exceeded limits for high data quality when using the WHO-GS. Reliability of measurements post-training was high as indicated by rTEMs below 1.5%. 3D imaging was highly correlated with manual measurements; however, on average 3D scans overestimated length and HC by 1.61 cm and 2.27 cm, respectively. Site staff preferred manual anthropometry to 3D imaging, as the imaging technology required adequate lighting and additional nuance when performing the measurements.

**CONCLUSIONS:** Manual anthropometry was feasible in presence of rigor mortis, and training improved digit preference. 3D imaging may be an accurate alternative to manual anthropometry, but technology adjustments are needed to ensure accuracy and usability. Future research on the appropriate use of current growth standards to define malnutrition in this severely ill population is needed.

**INTRODUCTION**

Malnutrition is estimated to contribute to approximately half of under-5-mortality (U5M) [1-3]. Malnutrition is also a major cause of morbidity as malnutrition plays a critical role in child neurodevelopment and health across the life course [2-4]. Reliable assessment tools for malnutrition are essential to reflect individual status, measure biological function, and predict health outcomes [5-7]. In children, inadequate growth is defined according to anthropometric measurements (length, weight, head and mid-upper arm circumference) that fall below 2 standard deviations of the normal sex-specific weight-for-length (wasting), length-for-age (stunting), and weight-for-age (underweight) [7]. Despite the importance of accurate anthropometry to detect early signs of malnutrition and monitor child growth, health facilities routinely use non-standardized anthropometric equipment, and as a result, measurements are often inaccurate [8]. Inaccurate measurements can lead to spurious classification of malnutrition in both individuals and populations[9].

In addition to the challenges of procuring and using standard anthropometric measurement tools, anthropometric measurements are subject to human error and are particularly difficult to collect among young children as children are easily distressed, have difficulty staying still, and may be unable to meet the requirements (i.e. ability to lie down or stand up) for manual anthropometry [10-12]. Anthropometric measurements are particularly challenging in hospitalized settings or in medically complex patients due to difficulty taking measurements due to IV’s, feeding tubes, severe illness, or limitations in mobility. These children are also at highest risk of malnutrition [8, 13]. Additionally, qualitative findings from a quality improvement study in a children’s hospital found that, wooden height-length measuring boards (ShorrBoard®, Weigh and Measure, LLC, Maryland USA) were considered to be “*heavy, cumbersome to assemble, frightening to patients, and required pre-planning and coordination between clinical staff with busy schedules and competing priorities*” [8]. Lastly, in field settings, the weight of the board may impede transportation and movement within the field and lack of standardization and maintenance of anthropometric equipment across study sites may contribute to poor data quality and misclassification [10, 11]. The post-mortem setting is another environment in which manual anthropometry may be challenging. Morgue capacity, rigor mortis, and edema can impact the quality and accuracy of measurements[14]. To our knowledge, no research has been conducted on the feasibility of using gold-standard anthropometric assessment in the postmortem setting.

The Child Health and Mortality Prevention Surveillance (CHAMPS) network is a multi-site surveillance system which strives to identify and understand the causes of under-5-mortality (U5M) in seven surveillance sites in sub-Saharan Africa and South Asia through detailed cause of death attribution with the use of high-quality postmortem anthropometrics, tissue samples, clinical abstraction, verbal autopsy, and the ability to integrate data from site-specific health and demographic surveillance systems (HDSS) [15, 16]. A recent analysis of the postmortem anthropometric data in CHAMPS suggested that nearly 90% of cases 1-59 months had evidence of undernutrition (stunting, wasting, or underweight) [17]. Given these data, it is possible that malnutrition is directly or indirectly associated with child mortality. However, our understanding of the relationship between malnutrition and mortality may also be hindered by poor anthropometric measurement data quality, including digit preference (e.g. measurement rounding), high percentage of biologically implausible values, and standard deviations for anthropometric indices that exceed acceptable limits, which may lead to misclassification of malnutrition [18-20]. These data quality and precision outcomes may be a result of shortages of standard equipment in CHAMPS sites, lack of training on manual anthropometry, or difficulty in conducting manual anthropometry in the postmortem setting (rigor mortis, poor lighting in morgue facilities).

Our primary objectives were to determine whether manual anthropometry is feasible in the postmortem setting and to quantify the impact of training and standard equipment on data quality. Given the practical challenges of performing manual anthropometry in field and hospital-based settings, various 3D imaging approaches have also been developed to obtain anthropometric measurements. An efficacy study conducted at Emory University found that a 3D imaging software was as accurate as gold-standard manual anthropometry among under-5 children in Atlanta-area daycare centers [10]. However, data are also needed to assess 3D imaging in challenging hospital- or field-based settings. Therefore, our secondary objective was to assess the validity and acceptability of 3D imaging for anthropometric assessment compared to gold-standard manual anthropometry.

**METHODS**

## This anthropometry study took place from October 2018 to September 2019 in the CHAMPS Manyatta, Kenya site located at the Jaramogi Oginga Odinga Teaching and Referral Hospital (JOOTRH). Prior to the training, site staff performed manual anthropometry on 75 deceased children as a routine part of the minimally invasive tissue sampling (MITS) portion of CHAMPS data collection.

## The MITS procedure is an abridged postmortem examination technique that has been validated for cause of death investigation in low-resource settings, described in detail elsewhere [21]. CHAMPS data collection procedures were approved by ethics committees in each CHAMPS site as well as Emory University (Emory IRB#: 00091706). Consent for MITS, verbal autopsy, and clinical data abstraction was obtained from parents or guardians. CHAMPS Ethical protocols are described in greater detail on the study website, ([https://champshealth.org/protocols/](https://nam11.safelinks.protection.outlook.com/?url=https%3A%2F%2Fchampshealth.org%2Fprotocols%2F&data=05%7C01%7Cpmgupta%40emory.edu%7C7d7f39520aed473da8d308dafae42741%7Ce004fb9cb0a4424fbcd0322606d5df38%7C0%7C0%7C638098155062477007%7CUnknown%7CTWFpbGZsb3d8eyJWIjoiMC4wLjAwMDAiLCJQIjoiV2luMzIiLCJBTiI6Ik1haWwiLCJXVCI6Mn0%3D%7C3000%7C%7C%7C&sdata=0ixyJfajDXZdcfqlV8uAf3k50CMigH7YxKZMIUhAjm0%3D&reserved=0)).

Upon conclusion of data collection, a senior nutritionist, pediatrician, and anthropometry expert led and conducted an on-site 1-week training on manual anthropometry and the 3D imaging scanner for 6 staff. The training on manual anthropometry emphasized best practices for accurate manual measures of length, weight, and circumference measurements using two trained anthropometrists and standard operating procedures [22]. Standard equipment in both sites, including wooden height-length measuring boards (ShorrBoard®, Weigh and Measure, LLC, Maryland USA), digital scales (Rice Lake Weighing Systems, Inc., Rice Lake, WI), and standard tape measures (Weigh and Measure LLC, Maryland USA), were used to ensure accurate measurement of recumbent length, weight, and head circumference (HC) and mid-upper arm circumference (MUAC), respectively. Staff were also trained on proper use the 3D imaging software; details on the imaging software are provided elsewhere [10, 23, 24]. Briefly, the AutoAnthro system uses an iPad^TM^ tablet, and a Structure Sensor^TM^ camera attached to the tablet to capture non-personally identifiable anthropometric scan images of the deceased child. Following the training, two unique site staff each performed manual anthropometry on 76 new cases, for a total of 2 manual measures per case. Additionally, 3D scans were completed in duplicate for each anthropometrist, for a total of 4 scans per case. Following data collection, it was found that the software settings had been inadvertently altered on the scanner resulting in viable scan data on only 23 cases.

***Outcomes of interest***

Key outcomes of interest included measures of data quality, precision, and accuracy. Data quality outcomes indicators included digit preference and standard deviations (SD) of anthropometric indices. Digit preference is the examination of a uniform distribution of terminal digits. We also calculated a digit preference score (DPS) to evaluate digit preference [25]. The DPS ranges from 0 to 100. Scores are low in instances of high agreement with the ideal of non-preference of the terminal digits, whereas DPS rises as the measures deviate from a uniform distribution across the terminal digits 0 through 9. In previous studies, a DPS cutoff above 20 was used to define the presence of digit preference [26, 27]. Previous studies have suggested acceptable standard deviation ranges specifically for data quality among living children [28]. These include 1.10-1.30, 1.00-1.20, 0.85-1.10 for length-for-age (HAZ), weight-for-age (WAZ), and weight-for-length (WLZ) z-scores, respectively. Z-scores for anthropometric indices were produced using the World Health Organization growth standards (WHO-GS) anthro R package [29].

Technical errors of measurement (TEM) were used to assess measurement precision. Following the training, the site staff performed manual anthropometry in duplicate. It is important to note that this differs from the data collection strategy pre-training in which a single set of measures were taken. As a result, we were only able to calculate TEMs for the data post-training in both sites. TEM express the error margin in anthropometry; they are unitless and allow comparison of errors across measures (e.g., weight, height etc.). Absolute TEMs were calculated using the formula outlined in Equation 1 (**Table 4**). Absolute TEMs can also be transformed into relative TEMs, which express the error as a percentage corresponding to the total average. Relative TEMs (rTEM) were calculated using the formula outlined in Equation 2 (**Table 4**). We used a cutoff of <1.5% rTEM to indicate a skillful anthropometrist [25].

Finally, Bland Altman plots were used to assess the accuracy of the 3D imaging software relative to manual anthropometry following the training and were quantified in the unit of the measure (cm or kg). Spearman correlation coefficients examined the strength of the relationship between scans and manual measures.

Following the study, a short survey was sent to the 6 study participants. The survey collected information on whether the participants believed training on manual anthropometry improved the accuracy of the measurements, whether 3D imaging reduced the time to measure, and asked about the participants preference in measuring using manual anthropometry or the 3D imaging technology. We also conducted a 60-minute in-depth interview with the single lead site technician to collect qualitative feedback on the team’s experience with performing manual anthropometry and ease of using the 3D imaging software. All analyses were conducted in RStudio [30].

We also conducted a small study in collaboration with the Pediatrics and Pathology departments at Children’s Healthcare of Atlanta, Egleston Hospital (CHOA). The goal was to evaluate whether manual anthropometry and 3D imaging performed consistently in a high-resource setting with adequate lighting and internet. The same training, detailed above, was used, and pathology staff notified the anthropometrists upon arrival of a case at the morgue. Manual anthropometry was to be performed prior to the start of the diagnostic autopsy. Significant challenges arose during data collection which resulted in a limited sample size of 3 cases; thus, our results will focus on the Kenya site.

**RESULTS**

Sample characteristics are summarized in **Table 1**. There were no significant differences in sample characteristics between the pre- and post- training groups. The majority of children were under 2 years of age, and were evenly distributed by sex. Proportions of stunting, wasting, and underweight were high, with a higher prevalence of stunting noted in the post-training group.

***Quality- Digit Preference***

In **Table 2**, prior to training, there was a clear tendency to round to the nearest 0.0 or 0.5 decimals for length, HC, and MUAC. There were no obvious signs of digit preference for weight measurement. The distribution of terminal digits post-training was evenly distributed for all measures. Similar patterns exist when examining the DPS. The DPS for length, pre-training, exceeded the acceptable limit, and post-training, the DPS for all measures fell below the acceptable cutoff of 20.

***Quality- Means and Standard Deviations of Anthropometric Indices***

**Table 3** summarizes the means and standard deviations for length-for-age (LAZ), weight-for-age (WAZ), and weight-for-length (WLZ), expressed as z scores. The standard deviations of all indices exceeded acceptable values both pre- and post-training. There were no differences in WAZ and WLZ pre- and post-training, but there was a statistically significant increase in LAZ post-training. There was a substantial loss in sample size when examining WLZ using WHO growth standards with 12% data loss in the pre- and 22% loss in the post-training group. This decrease in sample size when examining WLZ scores is due to nearly a fourth of the children having lengths below 45 cm or the smallest length captured by the WHO growth standards when calculating WLZ [31]. It is important to note that the WHO growth standards were based on a healthy population of children, receiving optimal nutrition, raised in optimal environments, and receiving optimal healthcare - unlike the cases captured in CHAMPS. Many of the CHAMPS cases, at the end of life, had attained sizes that are more comparable to growth and nutritional status in utero and may explain why CHAMPS cases are not compatible with postnatal life and survival at their chronologic age. There were no significant changes between the SDs for LAZ and WAZ pre- and post-training overall, and when stratified by age (<1 month vs 1-59 months as well as <6 months vs 6-59 months, data not shown).

***Precision-Technical Errors of measurement***

**Table 4** presents the TEMs and rTEMs specific to the post-training measures.

The TEMs for length, weight, HC, and MUAC were, 0.32, 0.01, 0.18, and 0.13 respectively. The rTEMs for length, weight, HC, and MUAC were 0.53%, 0.29%, 0.48%, and 1.24%, respectively. All TEMs and rTEMs were within the acceptable range.

***Accuracy- Spearman Correlation and Bland Altman Plots***

Spearman correlation coefficients (**Figure 1**) comparing the manual measures to the 3D scans for length, MUAC, and HC were 0.99, 0.91, and 0.93, respectively. While the manual measures were highly correlated with the scans, the mean differences between scans and manual measures for length, MUAC, and HC were 1.61 cm, -0.20 cm, and 2.27 cm, respectively. These results suggest that the scans overestimate length by 1.61 cm, underestimate MUAC by 0.20 cm, and overestimate HC by 2.27 cm.

While there were challenges in securing data at the CHOA site, findings were complementary to those in the Kenya site (data not shown). Among the 3 cases, standard anthropometry measurements were feasible and showed high precision (rTEMs for manual length, MUAC, and HC were 0.62%, 0.96%, and 1.80% respectively). For 3D scans, precision for duplicate scans was within acceptable limits when measuring length (rTEM=1.05%), but the software had more difficulty capturing precise measurements for MUAC (rTEM=4.71%) and HC (rTEM=1.62%).

***Qualitative findings: Use of 3D imaging in morgue setting***

The qualitative findings from the in-depth interviews revealed that the team had a clear preference for manual anthropometry over the 3D imaging software as they felt the 3D imaging software required more time, nuance (better lighting and improved morgue environment), and training to ensure an accurate scan.

*“We would take manual anthropometric measurements more seriously and would choose it well over 3D scanning...A lot of movement and manipulation of the camera to capture the entire body. And many times for 3D imaging, you have to repeat the process over and over and over again for you to be able to get the entire body into the screen. So it takes quite a bit more time…The boards work really well for us. It’s a stable board… it’s something we opt for over any other methods.”*

Additionally, study investigators cited challenges in using the software when lighting was insufficient or when morgue environments varied.

*“For what we experienced on the 3D, we had a few issues … our autopsy table had a fixed length and was not adjustable, so it was hard to get the complete image as you scan. Many times, we had issues with lighting systems. This made us end up with cut images—images with some parts of the body missing. So that called for checking and re-checking of images for quite a long period of time.”*

Lastly, study investigators noted postmortem-specific challenges to manual anthropometry and understood the implications for data quality and measurement error if careful measurement and attention to detail was not prioritized.

*“With rigor mortis, you will find that children stiffening, even the legs stiffening in some specific direction. If you are not able to manipulate them properly, one will end up with increased length as opposed to getting the accurate length. So that also required a lot of keenness.”*

*“the challenge in checking MUAC with tape measure comes when the subject you are measuring has reduced skin turgor. That is the skin of the arm becomes floppy. So that one might give you a lesser MUAC.”*

**DISCUSSION**

Following training on manual anthropometry and use of standard equipment for post-mortem assessment of nutritional status, data quality and precision were high, however, standard deviations of anthropometric indices exceeded acceptable values. 3D imaging scans overestimated length by approximately 1.6 cm, underestimated MUAC by 0.2 cm, and overestimated HC by 2.3 cm. The presence of rigor mortis did not impede the collection or quality of length measurements; however, additional care and pressure are critical to ensuring high quality data.

There was no evidence of digit preference for weight pre- or post-training, which is likely due to how the measurements were taken. Weight was read from a digital scale, while length and circumference measurements were reliant on the anthropometrist’s ability to use the equipment properly and read a tape measure accurately. Previous studies among living children have shown that the SD of anthropometric z-scores are reasonably consistent across populations, irrespective of nutritional status, and thus can be used to assess the quality of anthropometric data [32]. In Kenya, the SD for all anthropometric indices exceeded acceptable limits both pre- and post-training, and sensitivity analyses revealed that high SDs for LAZ and WAZ were unlikely to be explained by age. If we continue with the conclusion that the intervention may have contributed to high data quality and precision, then the persistently high SDs may be explained by capturing anthropometric measurements of small, severely ill children.

We also noted a decrease in sample size when examining WLZ scores. This is because nearly one-fourth of children in this sample fell below 45 cm, or the smallest length captured by the WHO growth standards when calculating WLZ [31]. It is important to note that the WHO growth standards were based on a healthy population of children, receiving optimal nutrition, raised in optimal environments, and receiving optimal healthcare - unlike the cases captured in CHAMPS. Many of the CHAMPS cases, at the end of life, had attained sizes that are more comparable to growth and nutritional status in utero and may explain why CHAMPS cases are not compatible with postnatal life and survival at their chronologic age. Future research might consider application of the INTERGROWTH-21 (IG21-GS) standards [33] to classify nutritional status of children that fall outside of the WHO-GS, such as in the case of CHAMPS enrolled cases. Comparing cases classified using the WHO-GS versus IG21-GS would enables us to understand how these children would rank, had they had survived.

This study has multiple strengths. First, to our knowledge, no research has been conducted on the feasibility of using gold-standard anthropometric assessment in the postmortem setting. Assessment of malnutrition and standardization of growth within the field of nutrition is typically based on z-scores derived from the 2006 WHO’s Multicentre Growth Reference Study (MGRS). These standards are based on healthy, living children, whereas being severely ill does not have a sufficient comparison group based on anthropometry. CHAMPS is a large, multi-site surveillance system, designed to elucidate the causes of U5M in high mortality regions of the world, therefore these standardized anthropometric data may help inform the possible ranges of anthropometric deficits in severely ill populations. Second, our project captured staff reflections and criticisms of conducting manual anthropometry in field-based and clinical-morgue settings. These qualitative findings may prove useful in informing strategies to improve accurate anthropometry in field-based and clinical-morgue settings given the structural and practical constraints of the environment.

This project was also subject to several limitations. First, in the CHOA site, we encountered unexpected obstacles in reaching our goal sample size. We learned that not all deceased children undergo autopsy and not all cases are routed to the morgue via the pathology department. When cases were routed to the morgue, there was limited time to conduct standard anthropometry and 3D imaging in duplicate or before autopsies were performed. Second, the need for two anthropometrists to arrive at the morgue and collect data before autopsy placed a significant burden on clinical staff and led to disruption of workflow. It should be noted, that within the CHOA site, autopsies are performed quickly and, in a step-wise fashion following the death of the child. There was often little time to balance case notification, standard equipment assembly and repeated measures. These challenges explain the limited sample size. Additionally, we found the pathologists were reluctant to using the 3D imaging software and the standard equipment. It appeared that knowledge of the importance of standard equipment was limited, although many had been introduced to the equipment earlier in their professional training. In the CHOA morgues, standard practice for securing postmortem measurements involved use of a tape measure, any deviations to this norm were resisted and were assumed to require additional time. Third, in Kenya, challenges arose with the 3D imaging software. The software settings were subject to user error and were altered during data collection, which resulted in a compromised final sample size. Among the viable scans, our results suggest that the scans overestimated both length and HC. These findings are aligned with a recent study [24] and further suggest that before 3D imaging can be considered a viable, accurate alternative to manual anthropometry, adjustment of the technology and additional user testing is warranted to ensure reliable anthropometric measures.

Collection of quality anthropometric data and implementation of standardized training and equipment is feasible in population-based, postmortem, field studies. Future research on the appropriate use of standards to define malnutrition among severely ill populations will elucidate our understanding of the role of malnutrition in U5M and inform future malnutrition-specific U5M reduction interventions.

**ACKNOWLEDGEMENTS**

The Child Health and Mortality Prevention Surveillance network would like to extend sincere appreciation to all the families who participated. Additionally, special thanks to Afrin Jahan for her analysis replication and figure development of Child Health and Mortality Prevention Surveillance (CHAMPS) network data related to this work.

**AUTHOR CONTRIBUTIONS**

PSS, PMG, KS, and JK designed and conducted the research. PMG analyzed the data, lead the manuscript development, and is primarily responsible for the final content. All authors have read and approved the final manuscript.

**REFERENCES**

1.	*Children: improving survival and well-being*. 2020; Available from: <https://www.who.int/news-room/fact-sheets/detail/children-reducing-mortality>.

2.	Pelletier, D.L., et al., *The effects of malnutrition on child mortality in developing countries.* Bull World Health Organ, 1995. **73**(4): p. 443-8.

3.	Rice, A.L., et al., *Malnutrition as an underlying cause of childhood deaths associated with infectious diseases in developing countries.* Bull World Health Organ, 2000. **78**(10): p. 1207-21.

4.	Schroeder, D.G. and K.H. Brown, *Nutritional status as a predictor of child survival: summarizing the association and quantifying its global impact.* Bull World Health Organ, 1994. **72**(4): p. 569-79.

5.	Salzberg, N.T., et al., *Mortality Surveillance Methods to Identify and Characterize Deaths in Child Health and Mortality Prevention Surveillance Network Sites.* Clin Infect Dis, 2019. **69**(Suppl 4): p. S262-s273.

6.	Suchdev, P.S., et al., *Assessment of Neurodevelopment, Nutrition, and Inflammation From Fetal Life to Adolescence in Low-Resource Settings.* Pediatrics, 2017. **139**(Suppl 1): p. S23-s37.

7.	Cashin, K., Oot, L.,. *Guide to Anthropometry: A Practical Tool for Program Planners, Managers, and Implementers*. 2018; Available from: <https://www.fantaproject.org/tools/anthropometry-guide>.

8.	Gupta, P.M., et al., *Improving assessment of child growth in a pediatric hospital setting.* BMC Pediatr, 2020. **20**(1): p. 419.

9.	Wit, J.M., et al., *Practical Application of Linear Growth Measurements in Clinical Research in Low- and Middle-Income Countries.* Horm Res Paediatr, 2017. **88**(1): p. 79-90.

10.	Conkle, J., et al., *Improving the quality of child anthropometry: Manual anthropometry in the Body Imaging for Nutritional Assessment Study (BINA).* PLoS One, 2017. **12**(12): p. e0189332.

11.	Mwangome M, B.J., *Measuring infants aged below 6 months: experience from the field.* Field Exchange, 2014(47).

12.	Bilukha, O., et al., *Comparison of anthropometric data quality in children aged 6-23 and 24-59 months: lessons from population-representative surveys from humanitarian settings.* BMC Nutrition, 2020. **6**(1).

13.	Sachdeva, S., et al., *Mid-upper arm circumference v. weight-for-height Z-score for predicting mortality in hospitalized children under 5 years of age.* Public Health Nutr, 2016. **19**(14): p. 2513-20.

14.	McCormack, C.A., et al., *Reliability of body size measurements obtained at autopsy: impact on the pathologic assessment of the heart.* Forensic Sci Med Pathol, 2016. **12**(2): p. 139-45.

15.	Raghunathan, P.L., S.A. Madhi, and R.F. Breiman, *Illuminating Child Mortality: Discovering Why Children Die.* Clin Infect Dis, 2019. **69**(Suppl 4): p. S257-s259.

16.	Blau, D.M., et al., *Overview and Development of the Child Health and Mortality Prevention Surveillance Determination of Cause of Death (DeCoDe) Process and DeCoDe Diagnosis Standards.* Clin Infect Dis, 2019. **69**(Suppl 4): p. S333-s341.

17.	Mehta, A., *Abstract: Prevalence of undernutrition among children 1-59 months in the Child Health and Mortality Prevention Surveillance (CHAMPS) Network*, in *The 22nd International Union of Nutritional Sciences (IUNS)-International Congress of Nutrition (ICN)*. 2022: Tokyo, Japan.

18.	Perumal, N., et al., *Anthropometric data quality assessment in multisurvey studies of child growth.* Am J Clin Nutr, 2020. **112**(Suppl 2): p. 806s-815s.

19.	Corsi, D.J., J.M. Perkins, and S.V. Subramanian, *Child anthropometry data quality from Demographic and Health Surveys, Multiple Indicator Cluster Surveys, and National Nutrition Surveys in the West Central Africa region: are we comparing apples and oranges?* Glob Health Action, 2017. **10**(1): p. 1328185.

20.	Prudhon, C., et al., *An algorithm to assess methodological quality of nutrition and mortality cross-sectional surveys: development and application to surveys conducted in Darfur, Sudan.* Popul Health Metr, 2011. **9**(1): p. 57.

21.	Rakislova, N., et al., *Standardization of Minimally Invasive Tissue Sampling Specimen Collection and Pathology Training for the Child Health and Mortality Prevention Surveillance Network.* Clinical Infectious Diseases, 2019. **69**(Supplement_4): p. S302-S310.

22.	Centers for Disease Control and Prevention, W.H.O., Nutrition International, UNICEF,. *Micronutrient survey manual*. 2020; Licence: CC BY-NC SA 3.0 IGO:[Available from: <https://mnsurvey.nutritionintl.org/>.

23.	Jefferds, M.E.D., et al., *Acceptability and Experiences with the Use of 3D Scans to Measure Anthropometry of Young Children in Surveys and Surveillance Systems from the Perspective of Field Teams and Caregivers.* Current Developments in Nutrition, 2022. **6**(6).

24.	Bougma, K., et al., *Accuracy of a handheld 3D imaging system for child anthropometric measurements in population-based household surveys and surveillance platforms: an effectiveness validation study in Guatemala, Kenya, and China.* The American Journal of Clinical Nutrition, 2022. **116**(1): p. 97-110.

25.	Oliveira, T., et al., *Technical error of measurement in anthropometry (English version).* Revista Brasileira de Medicina do Esporte, 2005. **11**: p. 81-85.

26.	SMART Action Against Hunger—Canada and Technical Advisory Group, *The SMART Plausibility Check for Anthropometry.* 2015.

27.	Conkle, J., et al., *Improving the quality of child anthropometry: Manual anthropometry in the Body Imaging for Nutritional Assessment Study (BINA).* PloS one, 2017. **12**(12): p. e0189332-e0189332.

28.	Mei, Z. and L.M. Grummer-Strawn, *Standard deviation of anthropometric Z-scores as a data quality assessment tool using the 2006 WHO growth standards: a cross country analysis.* Bull World Health Organ, 2007. **85**(6): p. 441-8.

29.	Dirk Schumacher, *anthro: Computation of the WHO Child Growth Standards*. 2021.

30.	RStudioTeam., *RStudio: Integrated Development Environment for R. RStudio, PBC,*. 2022: Boston, MA URL.

31.	*WHO Child Growth Standards based on length/height, weight and age.* Acta Paediatr Suppl, 2006. **450**: p. 76-85.

32.	Mei, Z. and L.M. Grummer-Strawn, *Standard deviation of anthropometric Z-scores as a data quality assessment tool using the 2006 WHO growth standards: a cross country analysis.* Bulletin of the World Health Organization, 2007. **85**(6): p. 441-448.

33.	Papageorghiou, A.T., et al., *The INTERGROWTH-21(st) fetal growth standards: toward the global integration of pregnancy and pediatric care.* Am J Obstet Gynecol, 2018. **218**(2s): p. S630-s640.

34.	Ulijaszek, S.J. and D.A. Kerr, *Anthropometric measurement error and the assessment of nutritional status.* Br J Nutr, 1999. **82**(3): p. 165-77.

**TABLES**

| **Table 1.** Sample characteristics among pre- and post-intervention groups, CHAMPS Study, Manyatta, Kenya, October 2018 to September 2019 | | |  |
| --- | --- | --- | --- |
|  | **Pre-intervention, n=75** | **Post-intervention,**  **n=76** | **p-value^4^** |
|  | **n(%)** | |  |
| **Age category, n (%)** | | |  |
| <1 day | 15 (20.0) | 21 (27.6) | 0.4821 |
| 1 day – 5 months | 28 (37.3) | 20 (26.3) |  |
| 6 – 23 months | 23 (30.7) | 25 (32.89) |  |
| 24 – 59 months | 9 (12.0) | 10 (13.1) |  |
|  |  |  |  |
| **Sex, n (%)** | | |  |
| Female | 31 (41.3) | 35 (46.1) | 0.5589 |
|  |  |  |  |
| **Anthropometric measurements, mean (SD)** | | |  |
| Weight, kg | 5.0 (3.8) | 4.8 (3.5) | 0.7543 |
| Length, cm | 62.0 (18.0) | 60.0 (17.6) | 0.4899 |
| Head circumference (HC), cm | 39.0 (6.9) | 37.9 (7.4) | 0.3509 |
| Mid-Upper Arm Circumference (MUAC), cm | 11.0 (3.0) | 10.2 (3.0) | 0.1064 |
|  |  |  |  |
| **Nutritional status, n (%)** | | |  |
| Stunting (LAZ^1^<-2SD) | 24 (32.0) | 38 (50.0) | 0.0246 |
| Wasting (WLZ^2^<-2SD) | 58 (77.3) | 54 (71.2) | 0.3780 |
| Underweight (WAZ^3^<-2) | 40 (53.3) | 50 (65.8) | 0.1188 |
| ^1^ LAZ: Length-for-age z-score  ^2^ WLZ: Length-for-weight z-score  ^3^ WAZ: Weight-for-age z-score  ^4^ p-values calculated using Chi Sq tests or t-tests | | | |

| **Table 2.** Digit preference^1^ pre- and post-intervention, CHAMPS Study, Manyatta, Kenya, October 2018 to September 2019 | | | | | | | | |
| --- | --- | --- | --- | --- | --- | --- | --- | --- |
| **Pre-** **intervention, (N=75)**  **n(%)** | | | | | **Post- intervention, (N=76)**  **n(%)** | | | |
| **Length** | | **Weight** | **HC** | **MUAC** | **Length** | **Weight** | **HC** | **MUAC** |
| **0.0** | 65 (86.7) | 15 (20.0) | 57 (77.3) | 54 (72.0) | 3 (4.0) | 10 (13.2) | 5 (6.6) | 2 (2.6) |
| **0.1** | - | 2 (2.7) | - | - | 12 (15.6) | 8 (10.5) | 11 (14.5) | 18 (23.7) |
| **0.2** | - | 6 (8.0) | - | - | 7 (9.2) | 9 (11.8) | 9 (11.8) | 10 (13.2) |
| **0.3** | - | 9 (12.0) | - | - | 13 (17.1) | 4 (5.3) | 3 (3.9) | 9 (11.8) |
| **0.4** | - | 6 (8.0) | - | - | 5 (6.6) | 9 (11.8) | 9 (11.8) | 5 (6.6) |
| **0.5** | 10 (13.3) | 6 (8.0) | 17 (22.7) | 21 (28.0) | 6 (7.9) | 9 (11.8) | 8 (10.5) | 9 (11.8) |
| **0.6** | - | 11 (14.7) | - | - | 7 (9.2) | 10 (13.2) | 13 (17.1) | 5 (6.6) |
| **0.7** | - | 9 (12.0) | - | - | 8 (10.5) | 4 (5.3) | 1 (1.3) | 5 (6.6) |
| **0.8** | - | 5 (6.7) | - | - | 8 (10.5) | 6 (7.9) | 12 (15.8) | 8 (10.5) |
| **0.9** | - | 6 (8.0) | - | - | 7 (9.2) | 7 (9.2) | 5 (6.6) | 5 (6.6) |
|  |  |  |  |  |  |  |  |  |
| **Digit preference score ^1^** | 86.2 | 15.3 | 78.1 | 74.3 | 10.4 | 9.5 | 16.6 | 18.4 |
| ^1^ Digit preference scores (DPS) computed using Mark Myatt and Ernest Guevarra (2022).  ^2^ nipnTK: National Information Platforms for Nutrition  Anthropometric Data Toolkit. https://nutriverse.io/nipnTK/,  <https://github.com/nutriverse/nipnTK>  DPS<20 is acceptable; ≥20 indicates digit preference is problematic | | | | | | | | |

| **Table 3.** Means and standard deviations for anthropometric indices, CHAMPS Study, Manyatta, Kenya, October 2018 to September 2019 | | | | | | |
| --- | --- | --- | --- | --- | --- | --- |
|  | **Pre-training, (N=75)** | | **Post-training, (N=76)** | | **p-value^1^** | **Expected SD for high data quality ^[28]^** |
|  | n | Mean (SD) | n | Mean (SD) |  |  |
| **LAZ^2^ overall** | 75 | -1.1 (2.6) | 76 | -2.5 (2.9) | 0.0018 | **1.1 – 1.3** |
| **< 1 months** | 35 | -0.8 (2.8) | 31 | -3.0 (3.2) |  |  |
| **1-59 months** | 40 | -1.4 (2.3) | 45 | -2.2 (2.7) |  |  |
|  |  |  |  |  |  |  |
| **WAZ^3^ overall** | 75 | -2.6 (2.3) | 76 | -3.2 (2.4) | 0.0962 | **1.0 – 1.2** |
| **< 1 months** | 35 | -2.0 (2.2) | 31 | -2.9 (2.2) |  |  |
| **1-59 m months** | 40 | -3.1 (2.3) | 45 | -3.5 (2.5) |  |  |
|  |  |  |  |  |  |  |
| **WLZ^4^ overall** | 66 | -3.1 (1.8) | 59 | -2.9 (2.2) | 0.4777 | **0.85 – 1.1** |
| **< 1 months** | 28 | -2.6 (1.1) | 15 | -1.5 (1.3) |  |  |
| **1-59 months** | 38 | -3.5 (2.1) | 44 | -3.3 (2.3) |  |  |
| ^1^ p-values comparing overall pre- and post-training mean z-scores calculated using t-tests  ^2^ LAZ: Length-for-age z-score  ^3^ WLZ: Length-for-weight z-score  ^4^ WAZ: Weight-for-age z-score | | | | | | |

| **Table 4.** Technical errors of measurement for post-intervention measures, CHAMPS Study, Manyatta, Kenya, October 2018 to September 2019 | | | | |
| --- | --- | --- | --- | --- |
|  | **Length (cm)** | **Weight (kg)** | **Mid-Upper Arm Circumference (cm)** | **Head Circumference (cm)** |
| **TEM^A^** | 0.32 | 0.01 | 0.13 | 0.18 |
| **Acceptable TEM ^[34]^** | 0.35 | 0.17 | 0.26 | - |
|  |  |  |  |  |
| **VAV** | 60.00 | 4.84 | 10.22 | 37.88 |
| **Relative TEM (% TEM)^c^** | 0.53% | 0.29% | 1.24% | 0.48% |
|  |  |  |  |  |
| The technical error of measurement (TEM) is defined as the standard deviation of differences between repeated measures in the unit of the measurement, using the following equation  ^A^ Equation 1: absolute technical errors of measurement (TEM) = $\sqrt{\frac{{\Sigma d_{i}}^{2}}{2n}}$  Where:  ${\Sigma d_{i}}^{2}$ = Squared summation of deviations, n = number of individuals measured, and i = number of deviations  ^C^ Equation 2: relative TEM =$100 x \frac{TEM}{VAV}$  Where TEM = technical error of measurement expressed as %, VAV= variable average value, the relative TEM (%TEM), and the coefficient of reliability (R) were the statistical tests used to assess intra- and inter-observer reliability. The TEM was defined as the standard deviation of differences between repeated measures in the unit of the measurement (e.g., TEM for height measured in centimeters is cm), using the following equation:  Skillful anthropometrists relative technical errors of measurement (%TEM) cutoff ≤ 1.5%  [25] | | | | |
