# Supplementary material for: Impact of anthropometry training and feasibility of 3D imaging on anthropometry data quality among children under five years in a postmortem setting
Source: PLoS One. 2023 Sep 28;18(9):e0292046. doi: 10.1371/journal.pone.0292046 (PMC10538800; doi:10.1371/journal.pone.0292046)
Supplement: Attachment — Submitted filename: PlosOne response to reviewers.docx [file pone.0292046.s005.docx]

**Response to Reviewers: PONE-D-23-01635**

Thank you for the thoughtful and detailed review of our manuscript. We have carefully revised and responded to each point raised by the academic editor and reviewers noted below in red font. We have also submitted a revised manuscript in track changes, as well as an unmarked revised manuscript.

Editor comments

1. Please ensure that your manuscript meets PLOS ONE's style requirements, including those for file naming. The PLOS ONE style templates can be found at

[https://journals.plos.org/plosone/s/file?id=wjVg/PLOSOne_formatting_sample_main_body.pdf](https://nam11.safelinks.protection.outlook.com/?url=https%3A%2F%2Fjournals.plos.org%2Fplosone%2Fs%2Ffile%3Fid%3DwjVg%2FPLOSOne_formatting_sample_main_body.pdf&data=05%7C01%7Cpsuchde%40emory.edu%7C6a2d9bc0c0e34e29fc1108db4f8ad298%7Ce004fb9cb0a4424fbcd0322606d5df38%7C0%7C0%7C638191230734862019%7CUnknown%7CTWFpbGZsb3d8eyJWIjoiMC4wLjAwMDAiLCJQIjoiV2luMzIiLCJBTiI6Ik1haWwiLCJXVCI6Mn0%3D%7C3000%7C%7C%7C&sdata=VnFiuhuLcgn1oghGdF%2B5eAKw28dsgfYFUJ2aCOphyB4%3D&reserved=0) and 
[https://journals.plos.org/plosone/s/file?id=ba62/PLOSOne_formatting_sample_title_authors_affiliations.pdf](https://nam11.safelinks.protection.outlook.com/?url=https%3A%2F%2Fjournals.plos.org%2Fplosone%2Fs%2Ffile%3Fid%3Dba62%2FPLOSOne_formatting_sample_title_authors_affiliations.pdf&data=05%7C01%7Cpsuchde%40emory.edu%7C6a2d9bc0c0e34e29fc1108db4f8ad298%7Ce004fb9cb0a4424fbcd0322606d5df38%7C0%7C0%7C638191230734862019%7CUnknown%7CTWFpbGZsb3d8eyJWIjoiMC4wLjAwMDAiLCJQIjoiV2luMzIiLCJBTiI6Ik1haWwiLCJXVCI6Mn0%3D%7C3000%7C%7C%7C&sdata=TObslrATdTQn07tI2JbLiuWBMEnXz8lv%2Br7o5RvTFSc%3D&reserved=0)

- Thanks we have made the required style changes including resubmitted the figure as a tiff file.

2. Please include a complete copy of PLOS’ questionnaire on inclusivity in global research in your revised manuscript. Our policy for research in this area aims to improve transparency in the reporting of research performed outside of researchers’ own country or community. The policy applies to researchers who have travelled to a different country to conduct research, research with Indigenous populations or their lands, and research on cultural artefacts. The questionnaire can also be requested at the journal’s discretion for any other submissions, even if these conditions are not met.

Please find more information on the policy and a link to download a blank copy of the questionnaire here: [https://journals.plos.org/plosone/s/best-practices-in-research-reporting](https://nam11.safelinks.protection.outlook.com/?url=https%3A%2F%2Fjournals.plos.org%2Fplosone%2Fs%2Fbest-practices-in-research-reporting&data=05%7C01%7Cpsuchde%40emory.edu%7C6a2d9bc0c0e34e29fc1108db4f8ad298%7Ce004fb9cb0a4424fbcd0322606d5df38%7C0%7C0%7C638191230734862019%7CUnknown%7CTWFpbGZsb3d8eyJWIjoiMC4wLjAwMDAiLCJQIjoiV2luMzIiLCJBTiI6Ik1haWwiLCJXVCI6Mn0%3D%7C3000%7C%7C%7C&sdata=mz34jSP2cyL24fft8ZCqmfzarNbKtmqnCk5AxDcflBA%3D&reserved=0).

Please upload a completed version of your questionnaire as Supporting Information when you resubmit your manuscript.

- Thanks we have completed and questionnaire and uploaded it as supporting information. We have also referred to the checklist in our Methods section

3.Please provide additional details regarding participant consent. In the ethics statement in the Methods and online submission information, please ensure that you have specified (1) whether consent was informed and (2) what type you obtained (for instance, written or verbal, and if verbal, how it was documented and witnessed). If your study included minors, state whether you obtained consent from parents or guardians. If the need for consent was waived by the ethics committee, please include this information.

If you are reporting a retrospective study of medical records or archived samples, please ensure that you have discussed whether all data were fully anonymized before you accessed them and/or whether the IRB or ethics committee waived the requirement for informed consent. If patients provided informed written consent to have data from their medical records used in research, please include this information.

- We have added 2 sentences in the Methods section describing both consent and study ethical clearance.

4. Thank you for stating the following in the Competing Interests section:

"I have read the journal's policy and the authors of this manuscript have the following competing interests:  Eugene Alexander holds an ownership position in Body Surface Translations and therefore has a financial interest in the success of the 3D testing device described in this study. Data were blinded and not shared with Mr. Alexander until completion of draft manuscript.

Additional disclosure: The findings and conclusions in this report are those of the authors and do not necessarily represent the official position of the Centers for Disease Control and Prevention."

Please confirm that this does not alter your adherence to all PLOS ONE policies on sharing data and materials, by including the following statement: ""This does not alter our adherence to  PLOS ONE policies on sharing data and materials.” (as detailed online in our guide for authors [http://journals.plos.org/plosone/s/competing-interests](https://nam11.safelinks.protection.outlook.com/?url=http%3A%2F%2Fjournals.plos.org%2Fplosone%2Fs%2Fcompeting-interests&data=05%7C01%7Cpsuchde%40emory.edu%7C6a2d9bc0c0e34e29fc1108db4f8ad298%7Ce004fb9cb0a4424fbcd0322606d5df38%7C0%7C0%7C638191230734862019%7CUnknown%7CTWFpbGZsb3d8eyJWIjoiMC4wLjAwMDAiLCJQIjoiV2luMzIiLCJBTiI6Ik1haWwiLCJXVCI6Mn0%3D%7C3000%7C%7C%7C&sdata=Fz4JIerMIcS4NVtQz%2FjVJdSM5KJ0OTF%2FT4KUGQPeydQ%3D&reserved=0)).

If there are restrictions on sharing of data and/or materials, please state these. Please note that we cannot proceed with consideration of your article until this information has been declared.

Please include your updated Competing Interests statement in your cover letter; we will change the online submission form on your behalf.

- Thanks, we have updated our competing interests statement to include “This does not alter our adherence to PLOS ONE policies on sharing data and materials.”

5. We note that you have indicated that data from this study are available upon request. PLOS only allows data to be available upon request if there are legal or ethical restrictions on sharing data publicly. For more information on unacceptable data access restrictions, please see [http://journals.plos.org/plosone/s/data-availability#loc-unacceptable-data-access-restrictions](https://nam11.safelinks.protection.outlook.com/?url=http%3A%2F%2Fjournals.plos.org%2Fplosone%2Fs%2Fdata-availability%23loc-unacceptable-data-access-restrictions&data=05%7C01%7Cpsuchde%40emory.edu%7C6a2d9bc0c0e34e29fc1108db4f8ad298%7Ce004fb9cb0a4424fbcd0322606d5df38%7C0%7C0%7C638191230734862019%7CUnknown%7CTWFpbGZsb3d8eyJWIjoiMC4wLjAwMDAiLCJQIjoiV2luMzIiLCJBTiI6Ik1haWwiLCJXVCI6Mn0%3D%7C3000%7C%7C%7C&sdata=z0UQKBkO7RIibH9T4W3F2JlRgdk3b5UF6v1SivFLkqY%3D&reserved=0).

In your revised cover letter, please address the following prompts:

a) If there are ethical or legal restrictions on sharing a de-identified data set, please explain them in detail (e.g., data contain potentially sensitive information, data are owned by a third-party organization, etc.) and who has imposed them (e.g., an ethics committee). Please also provide contact information for a data access committee, ethics committee, or other institutional body to which data requests may be sent.

b) If there are no restrictions, please upload the minimal anonymized data set necessary to replicate your study findings as either Supporting Information files or to a stable, public repository and provide us with the relevant URLs, DOIs, or accession numbers. For a list of acceptable repositories, please see [http://journals.plos.org/plosone/s/data-availability#loc-recommended-repositories](https://nam11.safelinks.protection.outlook.com/?url=http%3A%2F%2Fjournals.plos.org%2Fplosone%2Fs%2Fdata-availability%23loc-recommended-repositories&data=05%7C01%7Cpsuchde%40emory.edu%7C6a2d9bc0c0e34e29fc1108db4f8ad298%7Ce004fb9cb0a4424fbcd0322606d5df38%7C0%7C0%7C638191230734862019%7CUnknown%7CTWFpbGZsb3d8eyJWIjoiMC4wLjAwMDAiLCJQIjoiV2luMzIiLCJBTiI6Ik1haWwiLCJXVCI6Mn0%3D%7C3000%7C%7C%7C&sdata=Z9fxewsB1ZZIObNS1XQz4KiZmJwIIDE0N2Qp6Elpvzg%3D&reserved=0).

We will update your Data Availability statement on your behalf to reflect the information you provide.

- We have decided to share an anonymized dataset as Supporting Information files

6. We note that you have stated that you will provide repository information for your data at acceptance. Should your manuscript be accepted for publication, we will hold it until you provide the relevant accession numbers or DOIs necessary to access your data. If you wish to make changes to your Data Availability statement, please describe these changes in your cover letter and we will update your Data Availability statement to reflect the information you provide.

-As noted above, we have included our analysis datasets as a Supporting information file.

7. We note that you have included the phrase “data not shown” in your manuscript. Unfortunately, this does not meet our data sharing requirements. PLOS does not permit references to inaccessible data. We require that authors provide all relevant data within the paper, Supporting Information files, or in an acceptable, public repository. Please add a citation to support this phrase or upload the data that corresponds with these findings to a stable repository (such as Figshare or Dryad) and provide and URLs, DOIs, or accession numbers that may be used to access these data. Or, if the data are not a core part of the research being presented in your study, we ask that you remove the phrase that refers to these data.

- We have removed these statements as the data used are being shared as supporting information files

8. Please include a separate caption for figure in your manuscript.

- Done

Reviewers' comments:

Reviewer #1: The study is a very interesting study that will provide informaiton on how the errors in manual anthropometry can be improved in a post-mortem setting. Information claimed to be evaluated in the study will be very vital in nutrition assessment of children in post-mortem settings. However, the following observations were made during review:

1. The title of the article seem not to be approriate. Suggested title is presented in the comment section.

- Thanks for this feedback. Based on your and reviewer #2’s feedback, we have changed titled to “Impact of anthropometry training and feasibility of 3D imaging on anthropometry data quality among children under five years in a postmortem setting”

2. comments on the abstract, introduction and materials and methods, and results were made in the manuscript.

- Thank you for your suggestions. We have made all the suggested changes to the abstract, introduction, and methods sections.

3. Generally, authors seem not to define the objectives of the study properly and this is reflecting in the methodology, and result sections.

- We apologize for the confusion. We have tried to clearly define the objectives in the last paragraph of the introduction with both primary and secondary objectives. The methods and results section follow this same flow (e.g., presenting results of standard anthropometry data quality first, followed by study on 3D imaging)

4. Authors should interprete what is on the table correctly in the result section.
- We have reviewed and double-checked that all results in the table match their descriptions in the text.

5. The authors need to state the objectives of the study clearly and present results based on these objectives.

- please see response #3

6. Statistical analysis carried out was not clearly stated in the methodology. It is not appropriate to have to look at the table before having an idea of the statistics carried out.
- We have added a description of the statistical analyses conducted in the methods section

7. Figures indicated on the manuscript were not seen.
- We apologize- the figure was uploaded as a word document. It has now been resubmitted using the required tiff format

8. In the methodology, authors claimed to do a survey to collect information on whether the participants believed training on manual anthropometry improved the accuracy of the measurements, whether 3D imaging reduced the time to measure, and asked about the participants preference in measuring using manual anthropometry or the 3D imaging technology. In addition, authors also claimed to conducted a 60-minute in-depth interview with the single lead site technician to collect qualitative feedback on the team’s experience with performing manual anthropometry and ease of using the 3D imaging software. The results for these survey and qualitative study are not clear in the result sections as well as the tables.

Results were presented not indicating whether it is for manual anthropometry or 3D imaging (Tables 1-4). Although, I suppose that is for manual anthropometry. The results for pre- and post-training for the 3D scan were never presented and figure 1 was comparing manual anthropometry with 3 D imaging (although the figures were not seen).
Results on qualitative feedbacks were only presented for 3D imaging and not manual anthropometry.
- Thank you for this feedback. We have attempted to make the Methods and results section more clear. We have clarified table titles to indicate manual anthropometry, and have also changed the Figure title to clarify. We have also added additional text in the qualitative results section (lines 301-305).

9. Results on whether participants believed training on manual anthropometry improved the accuracy of the measurement or 3D imaging reduced the time to measure were not presented at all. Also, participants preference in measuring using manual anthropometry or 3D imaging technology was not presented in the result section.
- Thank you for raising this issue. We inadvertently excluded results from the post-training survey and have now added this to the “Qualitative findings” section of the results section (lines 300-305).

10. Discussion section needs to be re-written to reflect exactly what is in the results. In addition, results need to be properly discussed in line with findings from previous studies and implications should be discussed clearly and appropriately. Assumptions in the result sections is not approriate.

- Thanks for your comment. We have attempted to re-write sections of the Discussion section to improve clarity and insure that findings are consistent to what were reported in the Results.

11.Authors did not have conclusion section at all.

- The last paragraph was our conclusions, which has now been appropriately labeled. The wording of the Conclusion has also been updated.

12. There is need for proper organization of the content of the manuscripts for coherence.
- Thank you for your comment. We have added several new headers and text to improve the organization and flow of the manuscript

13. Some sentences seem complicated and difficult to understand. The authors are advised to seek for professional English editing service to check the revised manuscript for grammar, syntax and style errors.

- Thanks for your comment. We have made appropriate grammatical and style edits to improve clarity.

Reviewer #2: PONE-D-23-01635
This is an important topic because the findings add to the existing evidence on causes of death due to anthropometric deficits in children. This study is novel and a useful contribution to the body of evidence on child health and nutrition. The paper fits the PlosOne journal’s aim and will be interesting to your readership.

The analysis is comprehensive and accurate. Limitations and strengths of the analysis have been declared adequately. Data analysis and results were adequately done and well presented. This paper deserves to be published.

Please, find below suggested minor comments and suggestions for your consideration to further improve your manuscript:

Comments:
Title
1. The current title of the article should be modified to reflect manual anthropometry as well as 3D imaging and the target or study population - children under 5 years.

- Thank you for your feedback. We have changed title to “Impact of anthropometry training and feasibility of 3D imaging on anthropometry data quality among children under five years in a postmortem setting”

Abstract
1. Please, could the conclusion “Future research on the appropriate use of current growth standards to define malnutrition in this severely ill population is needed” be revised to reflect the topic of interest. …. This severely ill population is not very clear, I thought the study setting was post-mortem.

- Thank you. We have modified the conclusion in the abstract and removed the sentence on use of growth standards, as this requires additional explanation in the Discussion section of the manuscript.

Method
1. In paragraph 3 …. weight, and circumference measurements using two…. please could indicate which circumference measurement you are referring to?

- Thank you for your feedback. We have updated the text in line 162 to reflect that head and mid-upper arm circumference are the two circumference measurements.

2. Please revise the sentence in paragraph 3 …. ‘Following the training, two unique site staff each performed manual anthropometry on 76 new cases, for a total of 2 manual measures per case’ and make it simpler and clearer.

- Thank you for the feedback. We revised lines 174-154 to say “Following the training, two trained anthropometrists manually collected anthropometric measurements for 76 cases, with two separate measurements collected per case by different anthropometrists”.

3. Authors should check if the sentence in paragraph 3 “Following data collection, it was found that the software… settings had been inadvertently altered on the scanner resulting in viable scan data on only 23 cases.” is communicating the right message, because if the software was inadvertently altered then it could me the data was not viable. I may be wrong. If that was true, then how did it impact on the findings?

-Thank you for the feedback. We revised lines 177-178 to say “During data processing, after the completion of data collection, it was identified that the AutoAnthro software settings had been inadvertently altered for a significant number of cases, resulting in a final sample size of 23 cases”.

4. Clarity needed what actually happened? ……‘Manual anthropometry was to be performed prior to the start of the diagnostic autopsy. Significant challenges arose during data collection which resulted in a limited sample size of 3 cases; thus, our results will focus on the Kenya site’.

- Thank you for the feedback. We have clarified line 230 to say “Significant challenges arose during data collection, including identification of eligible cases and availability to conduct manual anthropometry before the start of the diagnostic autopsy. Despite best efforts to coordinate between the study team and CHAO team, the study resulted in a limited sample size of 3 cases; thus, our results will focus on the Kenya site.

5. What was the duration of the training? What was the duration of the data collection?

-The Kenya site training took place in April 2019 followed by data collection from April-September 2019. The training for the CHOA study took place in September 2019 with data collection in October-November 2019.

6. Please indicate how you analysed the qualitative data, and how you utilized the data.

-Thank you for the feedback. We added information on the statistical analysis of the qualitative data and how we utilized the qualitative data in lines 219-223.

Results

1. ‘There was a substantial loss in sample size when examining WLZ using WHO growth standards with 12% data loss in the pre- and 22% loss in the post-training group’.
How was LAZ also affected given that there was data lost for WLZ?
- Thanks for this question. When using the WHO growth standards, the absolute limit of 45cm in length only applies to the calculation of WLZ, not LAZ since LAZ is estimable because it is based on standardizing child length according to their completed age (and by sex). 45cm was determined by WHO as the minimum birth length for healthy children (e.g., without any intrauterine growth restriction and or congenital disorder of size).

2. Why will authors talk about results that are not available?
‘While there were challenges in securing data at the CHOA site, findings were complementary to those in the Kenya site (data not shown)’.
- In response to earlier comment by editor, we have deleted the statement “data not shown” and have shared datasets as supporting files.

Discussion

1. Authors, please explain why rigor mortis will not impede manual anthropometry measurements, this is because the qualitative findings show that it could be a challenge to get accurate measurements.

- Thanks for this comment. We have edited the qualitative findings in the results to make this more clear. While there were challenges in taking manual anthropometric measurements due to rigor mortis, the stiffening was always accounted for with added pressure and time. Thus, the accuracy of measurements was ensured using the wooden length boards.

2. Table 3 in the results section and Paragraph 3 in the discussion have some repetitions, this happened because you cited literature in your results section. Authors should consider to present only results under the results section.

-Thank you for the feedback. We removed the duplicated information from Table 3 in the results section.

3. Check WHO-GS should be written as WHO-MGRS.

-Thanks for this comment. We have written out WHO growth standards and WHO Multicentre Growth Reference Study when appropriate.

4. ‘Future research on the appropriate use of standards to define malnutrition among severely ill populations will elucidate our understanding of the role of malnutrition in U5M and inform future malnutrition-specific U5M reduction interventions’ I think I know what you are trying to say but I am wandering if this appropriate recommendation because you did not work with severely sick children. Please consider to revise your recommendation.
- Thanks for this comment. We have revised our concluding statement.
General comments

1. Please number the lines for the sentences in your manuscript. It helps reviewers to give feedback easily.

- Line numbers have been added
