# Supplementary material for: Impact of anthropometry training and feasibility of 3D imaging on anthropometry data quality among children under five years in a postmortem setting
Source: PLoS One. 2023 Sep 28;18(9):e0292046. doi: 10.1371/journal.pone.0292046 (PMC10538800; doi:10.1371/journal.pone.0292046)
Supplement: Attachment — Submitted filename: Reviewer report_2.docx [file pone.0292046.s006.docx]

**PONE-D-23-01635R1**

**Impact of anthropometry training and feasibility of 3D imaging on anthropometry data**

**quality among children under five years in a postmortem setting**

**Comments to the editor and authors**

I wish to thank the authors for considering my comments in the revision of this manuscript, however, I have one comment on how qualitative data was analysed. I have seen your response below. I wish you were more elaborate on how you analysed the qualitative data. I am concern because most of us researchers don’t pay keen attention to qualitative data especially when we are using both quantitative and qualitative methods in a study.

If the academic editor is satisfied with your response, then PLOS-One can go ahead with publication of the manuscript.

Thank you once again for the opportunity to review your paper.

**Comments:**

6. Please indicate how you analysed the qualitative data, and how you utilized the data.

-Thank you for the feedback. We added information on the statistical analysis of the qualitative data and how we utilized the qualitative data in lines 219-223.

Line 230-231 as found in the manuscript.

230 The qualitative data were utilized to improve the implementation of manual 231 anthropometric measurements across the CHAMPS Network.
