# Supplementary material for: Impact of anthropometry training and feasibility of 3D imaging on anthropometry data quality among children under five years in a postmortem setting
Source: PLoS One. 2023 Sep 28;18(9):e0292046. doi: 10.1371/journal.pone.0292046 (PMC10538800; doi:10.1371/journal.pone.0292046)
Supplement: Attachment — Submitted filename: PlosOne response to reviewers2.docx [file pone.0292046.s008.docx]

**Response to Reviewers: PONE-D-23-01635**

Thank you for the thoughtful review of our revised manuscript. We have carefully revised and responded to each point raised by the reviewers noted below in red font. We have also submitted a revised manuscript in track changes, as well as an unmarked revised manuscript.

**Reviewer #1**

The research design is not clearly stated. You need to state the design for the quantitative and the qualitative study. What design was used for the pre- and post- conducted. This is a very important.

- We have revised the first sentence of the Methods section to further clarify the research design, emphasizing the both quantitative and qualitative research approach was used.

“This longitudinal quality improvement study adopted a mixed-methods approach utilizing quantitative and qualitative research on the experience conducting manual anthropometry and 3D imaging in the postmortem setting.”

Kindly give a detailed information on the content of the interview.

- We believe we have adequately described the content of the interview on lines 205-206 as stated “…to collect qualitative feedback on the team’s experience with performing manual anthropometry and ease of using the 3D imaging software.”

The title of this result should be stated before the interpretation.

- We are unclear about this comment. Hopefully it is resolved with response to other comments about interpretation of Table 1 below.

It is better to report the improvement or otherwise that happened post-intervention.

- We have changed the order and wording in lines 226-231

There is significant differences in the sample characteristics for stunting (as shown in table 1). This should reflect in the result interpretation and the p-value should be stated. The type of statistics carried out should be clearly stated in the methodology.

- We have reworded the interpretation of findings in Table 1. The statistical approach is described in the Methods--“Pearson’s Chi-Square tests (categorical variables) or t-tests (continuous variables) were used to evaluate differences between pre-intervention and post-interventions groups-- and also in the legend of the table.

Digital Preference Score of the respondents anthropometric indices is suggested as title.

Digital preference score was used to assess respondents' anthropometric indices and that should reflect in the title

- We support keeping the subheading the same as we report both absolute digit values and calculated DPS in table 2. To further clarify, DPS are not related to calculation of anthropometric indices and simply reflect interpretations of the raw recorded anthropometric measurements.

Mean anthropomentry of the respondents. This result should come before the DPS

- As noted above, the DPS is calculated before entering the measurements into statistical software for calculation of anthropometric indices. This we support keeping table 3 after table 2.

Except the WLZ of the children of ages <1 month which was within the acceptable SD for high quality data.

- We appreciate this comment and have modified the text accordingly.

State the p value (e.g. at p<0.01) for where there is statistical difference.

- We have added the p-value noted in the table.

This statement is not correct. Kindly check your table clearly and ensure that what is on the table is in agreement with what is interpreted here in the result section.

This statement is not correct. Kindly check your table clearly and ensure that what is on the table is in agreement with what is interpreted here in the result section. There was rather increase in the WLZ

- We have moved up this sentence to avoid confusion and also added the sample size of number of children who dropped out when calculated WLZ (n=9 and n=17 in the pre- and post-training groups, respectively. The calculations of data loss are correct.

You cannot assume that it is due to how the measurements were taken. You need to discuss this in line with previous findings.

- Thanks for this comment. We explain the rationale for this statement in the following sentence. “Weight was read from a digital scale, while length and circumference measurements were reliant on the anthropometrist’s ability to read a tape measure accurately.”

**Reviewer #2**

I wish to thank the authors for considering my comments in the revision of this manuscript, however, I have one comment on how qualitative data was analysed. I have seen your response below. I wish you were more elaborate on how you analysed the qualitative data. I am concern because most of us researchers don’t pay keen attention to qualitative data especially when we are using both quantitative and qualitative methods in a study.

- We appreciate this request for additional description of the qualitative analysis and have revised the description as follows (line 210-212): “The qualitative data were analyzed using simple frequencies and applying manual thematic analysis; findings informed the implementation of manual anthropometric measurements across the CHAMPS Network.”
